# Chromosomal-level genome assembly of *Melastoma candidum* provides insights into trichome evolution

**DOI:** 10.3389/fpls.2023.1126319

**Published:** 2023-01-27

**Authors:** Yan Zhong, Wei Wu, Chenyu Sun, Peishan Zou, Ying Liu, Seping Dai, Renchao Zhou

**Affiliations:** ^1^ State Key Laboratory of Biocontrol and Guangdong Provincial Key Laboratory of Plant Resources, School of Life Sciences, Sun Yat-sen University, Guangzhou, China; ^2^ Guangzhou Institute of Forestry and Landscape Architecture, Guangzhou, China

**Keywords:** *Melastoma candidum*, genome assembly, trichome evolution, whole genome duplication, transcription factor

## Abstract

*Melastoma*, consisting of ~100 species diversified in tropical Asia and Oceania in the past 1-2 million years, represents an excellent example of rapid speciation in flowering plants. Trichomes on hypanthia, twigs and leaves vary markedly among species of this genus and are the most important diagnostic traits for species identification. These traits also play critical roles in contributing to differential adaptation of these species to their own habitats. Here we sequenced the genome of *M. candidum*, a common, erect-growing species from southern China, with the aim to provide genomic insights into trichome evolution in this genus. We generated a high-quality, chromosome-level genome assembly of *M. candidum*, with the genome size of 256.2 Mb and protein-coding gene number of 40,938. The gene families specific to, and significantly expanded in *Melastoma* are enriched for GO terms related to trichome initiation and differentiation. We provide evidence that *Melastoma* and its sister genus *Osbeckia* have undergone two whole genome duplications (WGDs) after the triplication event (γ) shared by all core eudicots. Preferential retention of trichome development-related transcription factor genes such as C2H2, bHLH, HD-ZIP, WRKY, and MYB after both WGDs might provide raw materials for trichome evolution and thus contribute to rapid species diversification in *Melastoma*. Our study provides candidate transcription factor genes related to trichome evolution in *Melastoma*, which can be used to evolutionary and functional studies of trichome diversification among species of this genus.

## 1 Introduction

**Figure 1:**
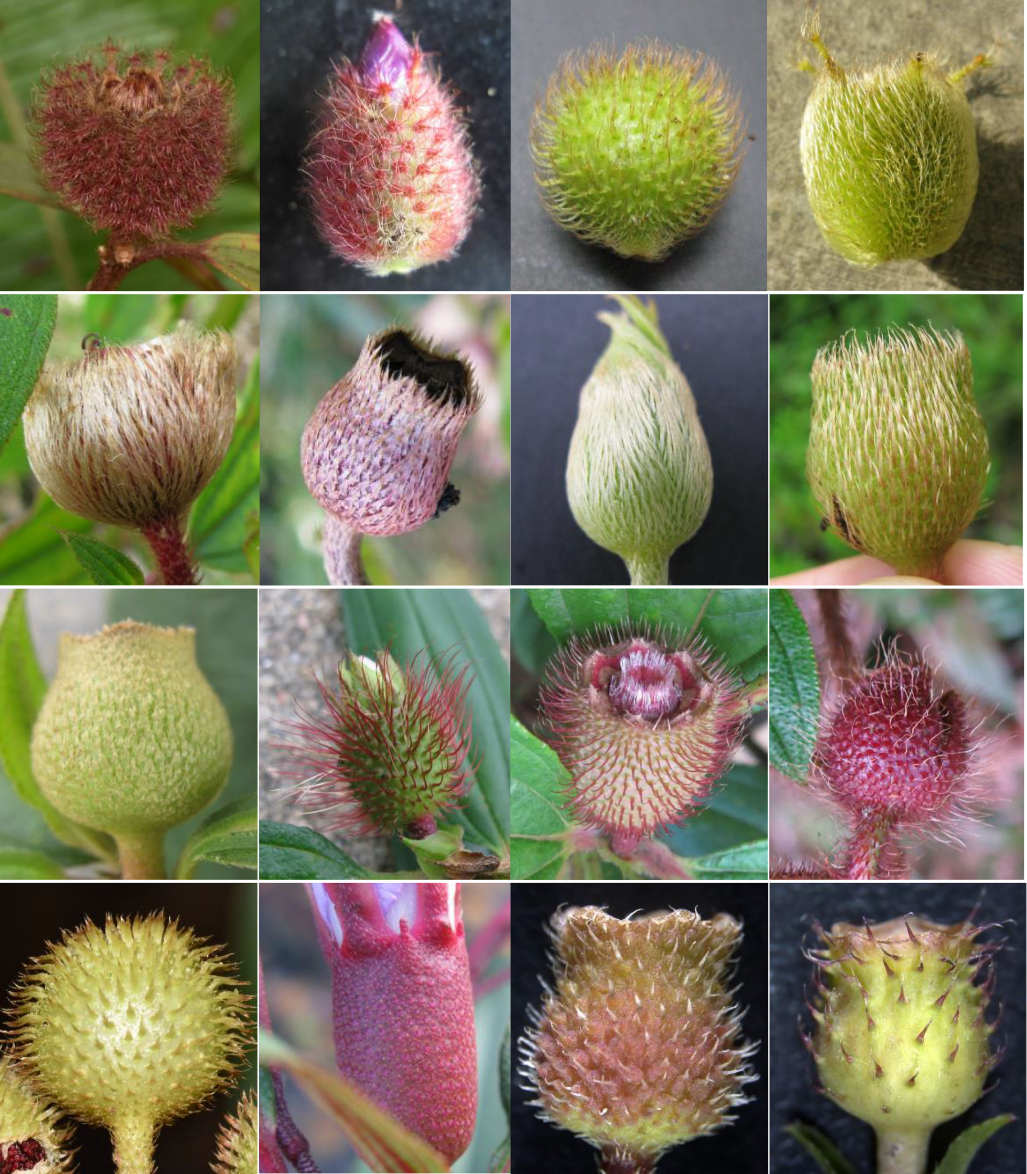
Trichomes on the hypanthia of *Melastoma*. From left to right, the first row: *Melastoma saigonense* (Vietnam), *M. beccarianum* (Malaysia), *M. dendrisetosum* (China), *M. ultramaficum* (Malaysia); the second row: *M. sabahense* (Malaysia), *M. normale* (China), *M. candidum* (China), *M. affine* (China),; the third row: *M. setigerum* (Indonesia), *M. sanguineum* (Cambodia), *M. sanguineum* (China), *M. penicillatum* (China); the fourth row: *M.* sp. (Vietnam), *M. laevifolium* (Malaysia), *M. kudoi* (China), *M. dodecandrum* (China).

*Melastoma* is a shrub genus distributed in tropical Asia and Oceania, with Southeast Asia as its species diversification center. This genus comprises about 100 species (Chen, 1984; Wong, 2016), which were estimated to be formed in the past 1-2 million years (Renner and Meyer, 2001), thus represents an exceptional example of rapid species diversification in plants. All species of *Melastoma* have an erect-growing habit except *M. dodecandrum*, which is the only creeping species and also the first diverging species in this genus (Dai et al., 2019). Species of *Melastoma* are mainly recognized by trichomes in the hypanthia, young stems and leaves, which show a very rich diversity in shape, size, density and color among species (Wong, 2016). For example, the trichomes on the hypanthia include stellate hairs, scales, bristles, soft hairs and so on (**Figure 1**).

Trichomes possess protective functions and defense mechanisms against biotic and abiotic stresses such as herbivores, pathogens, and ultraviolet (UV) irradiation (Kang et al., 2010; Riddick and Simmons, 2014; Bickford, 2016; Rakha et al., 2017). It also plays an important role in biological functions such as development, seed dispersal, adaptation to extreme temperatures, and signal transmission (Hegebarth et al., 2016; Zhao and Chen, 2016; Zhou et al., 2017). Previous studies in *Melastoma* suggested that trichomes of different species or populations might contribute to their differential adaptation to heterogenous habitats (Ng et al., 2019). For example, *M. candidum*, always found in open habitats, has densely covered scales in the hypanthia and densely covered hairs in the leaves, which can resist the (UV) irradiation. In contrast, *M. sanguineum* usually occurs in shady understory, has sparse bristles in its hypanthia and glabrous leaves (Liu et al., 2014; Ng et al., 2019). In *M. normale*, populations with red and white trichomes in the young stems (twigs) exhibit higher fitness in their own habitats with high and low sunlight intensities, respectively, indicating differential adaptation^1^. Therefore, trichomes appear to be a key trait in *Melastoma*, providing various ecological opportunities in facilitating rapid species diversification in this genus⁠. Under the ecology opportunity hypothesis (Schluter, 2000; Stankowski and Streisfeld, 2015), the ancestral species may have evolved some key ecologically related traits to take advantage of available resources.

A variety of factors, such as regulatory genes, non-coding RNAs, hormones and environment, are involved in regulating plant trichome initiation, growth and differentiation (Wang et al., 2019; Wang et al., 2021). Previous studies found that many transcription factors including R2R3-MYB, bHLH, WD40, HD-ZIP, WRKY and C2H2, play a critical role in trichome development of *Arabidopsis* and cotton (Yang and Ye, 2013; Wang et al., 2021). However, trichome development is regulated by different mechanisms in different plants, especially in the multicellular trichomes produced by most plants. For example, although the bHLH transcription factors are essential for the initiation of trichomes differentiation in *Arabidopsis*, it has no effect in tobacco (*Nicotiana tabacum*) and tomato (*Solanum lycopersicum*) (Lloyd et al., 1992).

Genes functioning in the initiation and differentiation of trichomes have been characterized in model plants like *Arabidopsis* (Szymanski et al., 2000), cotton (Zhao and Chen, 2016) and tomato (Rakha et al., 2017), but similar studies have been rarely conducted in non-model plants, including *Melastoma* in which trichomes play an important role in species diversification and ecological adaptation. Many genomic processes including whole genome duplication and gene family expansion can provide raw materials for the evolution of new traits and adaption to novel environments in plants (Li et al., 2016; Feng et al., 2020; Wu et al., 2020). Based on the remarkable trichome diversity in *Melastoma*, we predict that trichome-related genes might have been expanded in the genome of this genus. To date, only the genome of *M. dodecandrum* has been reported (Hao et al., 2022), however, the annotation of this genome is not complete (see Results) and no analyses on trichome evolution have been performed in that study. Here we report the sequencing, assembly, annotation and characterization of the genome of *M. candidum*, an erect-growing species widely distributed in southern China, northern Vietnam and Okinawa of Japan (Chen, 1984). We aimed to connect the genomic features in *Melastoma* and thus to understand trichome evolution in this genus.

## 2 Results

### 2.1 Genome assembly and annotation

**Figure 2:**
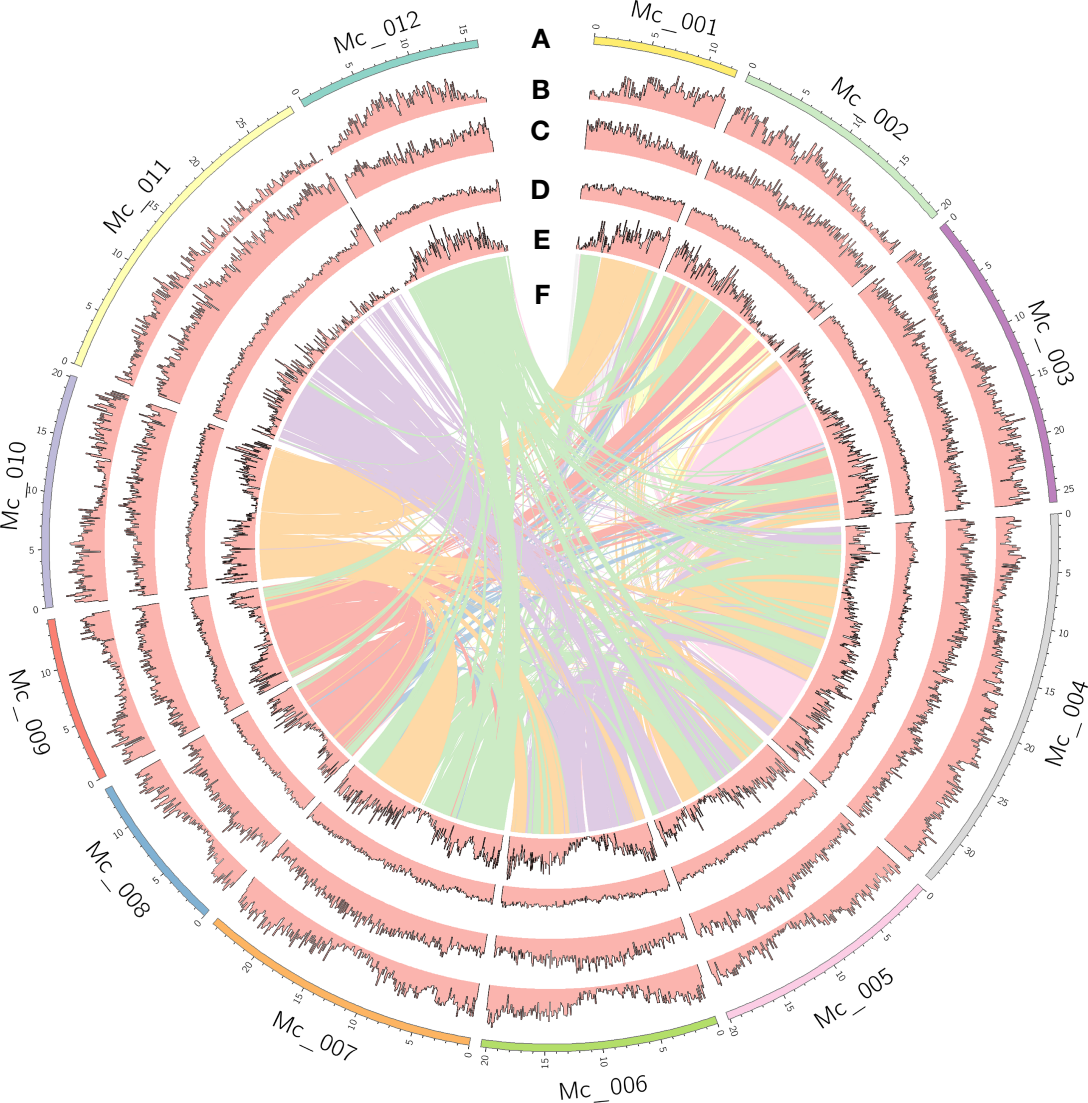
Genome features of *Melastoma candidum*. Tracks displayed are: **(A)** 12 pseudochromosomes; **(B)** gene density; **(C)** density of repeats; **(D)** GC content; **(E)** density of genes in the syntenic blocks; **(F)** inter-chromosome synteny.

The genome size of *M. candidum* was estimated to be about 257.1 Mb based on a K-mer (k=21) analysis of Illumina sequencing data (**Figure S1**). Using PacBio long reads, we generated a genome assembly of 256.2 Mb, which represents 99.7% of the estimated genome size and consists of 266 scaffolds. 98.0% (251.2 Mb) of the scaffold sequences were anchored to the 12 pseudochromosomes based on the Hi-C data (**Figure 2**). The N50 and N90 of the scaffolds were 20.5 Mb and 13.7 Mb, respectively (**Table 1**). The PacBio long reads and Illumina short reads have mapping rates of 96.0% and 97.9%, and cover 99.8% and 99.6% of the genome, respectively (**Table S1**). The total mapping rate of Illumina RNA-seq reads to the genome was 95.6% (**Table S1**). 96.9% of 1614 Benchmarking Universal Single-Copy Orthologs (BUSCOs) genes in the embryophyta_odb10 and 92.9% of 2326 BUSCOs genes in the eudicots_odb10 datasets were recovered in our genome assembly (**Table S2**). The LTR Assembly Index (LAI) across the genome is 25.5. All the genome continuity, completeness and accuracy assessment results above suggest that the genome assembly of *M. candidum* is of high quality.

**Table 1:** Summary of genome assembly and annotation for *Melastoma candidum*.

Assembly features	
Genome-sequencing depth (×)	212
Assembly genome size (Mb)	256.2
Estimated genome size (Mb)	257.1
GC content	42.9%
Scaffolds number	266
Scaffold N50 (bp)	20,460,156
Scaffold L50	5
Scaffold N90 (bp)	13,725,599
Scaffold L90	11
Contig N50 (bp)	2,018,067
Contig N90 (bp)	447,033
Annotation features	
Number of predicted genes	40,938
Mean gene length (bp)	2387.2
Mean exon length (bp)	278.9
Mean intron length (bp)	227.7
Mean of exon number per gene	5.2
Repeat content (% of the genome assembly)	80.6 Mb (31.5%)
Number of functionally annotated genes	37,393

N50: sequence length of the shortest contig/scaffold at 50% of the total genome length

L50: the smallest number of contigs/scaffolds whose length sum makes up half of genome size

N90: sequence length of the shortest contig/scaffold at 90% of the total genome length

L90: the smallest number of contigs/scaffolds whose length sum makes up 90% of genome size

Repetitive sequences account for 31.5% of the genome (**Table 1**). Most of them are long terminal repeat retrotransposons (LTR), covering 23.1% of the genome (**Table S3**). The two major superfamilies, Ty3/*Gypsy* and Ty1/*Copia*, account for 11.7% and 7.8% of the genome, respectively. The DNA transposons take up 6.3% of the genome. We predicted 40,938 protein-coding genes in the *M. candidum* genome (**Table 1**), by combining *de novo* prediction, transcriptome evidence and homology-based approaches. 91.3% genes could be annotated in at least one of the functional annotation databases (**Table 1**; **Table S4**). The average exon and intron sizes were 279 bp and 228 bp, respectively (**Table 1**). In addition, 1,818 non-coding RNAs including 188 miRNAs, 233 rRNAs, 699 tRNAs, and 698 snRNAs were identified. 96.0% and 93.5% of the BUSCOs genes in the two datasets mentioned above were recovered based on our genome annotation (**Table S5**).

Although the BUSCO assessment revealed comparable gene recovery rates between the genomes of *M. candidum* and *M. dodecandrum*, we found that the *M. candidum* genome has higher proportion of single-copy genes (72.4%) and lower proportion of duplicated genes (20.5%) of complete BUSCOs than the *M. dodecandrum* genome (69.4% and 23.9%, respectively) in the eudicots_odb10 dataset (**Figure S2**). Based on the genome annotation, *M. candidum* has 5,257 (12.8% of the predicted 40,938 genes) more genes than *M. dodecandrum*, in which 35,681 genes were predicted. Meanwhile, the BUSCO assessment with protein mode showed that, of the 2,326 genes in the eudicots_odb10 dataset, *M. candidum* recovered 231 more genes than *M. dodecandrum* (**Figure S2**). The 231 genes are either fragmented (60) or missing (171) in *M. dodecandrum*. The similar situation was observed in the embryophyta_odb10 dataset (**Figure S3**). Taken together, this suggests incomplete gene annotation for the *M. dodecandrum* genome, given very low divergence between the two species (see below).

### 2.2 Phylogenetic position and short species diversification history of *Melastoma*

**Figure 3:**
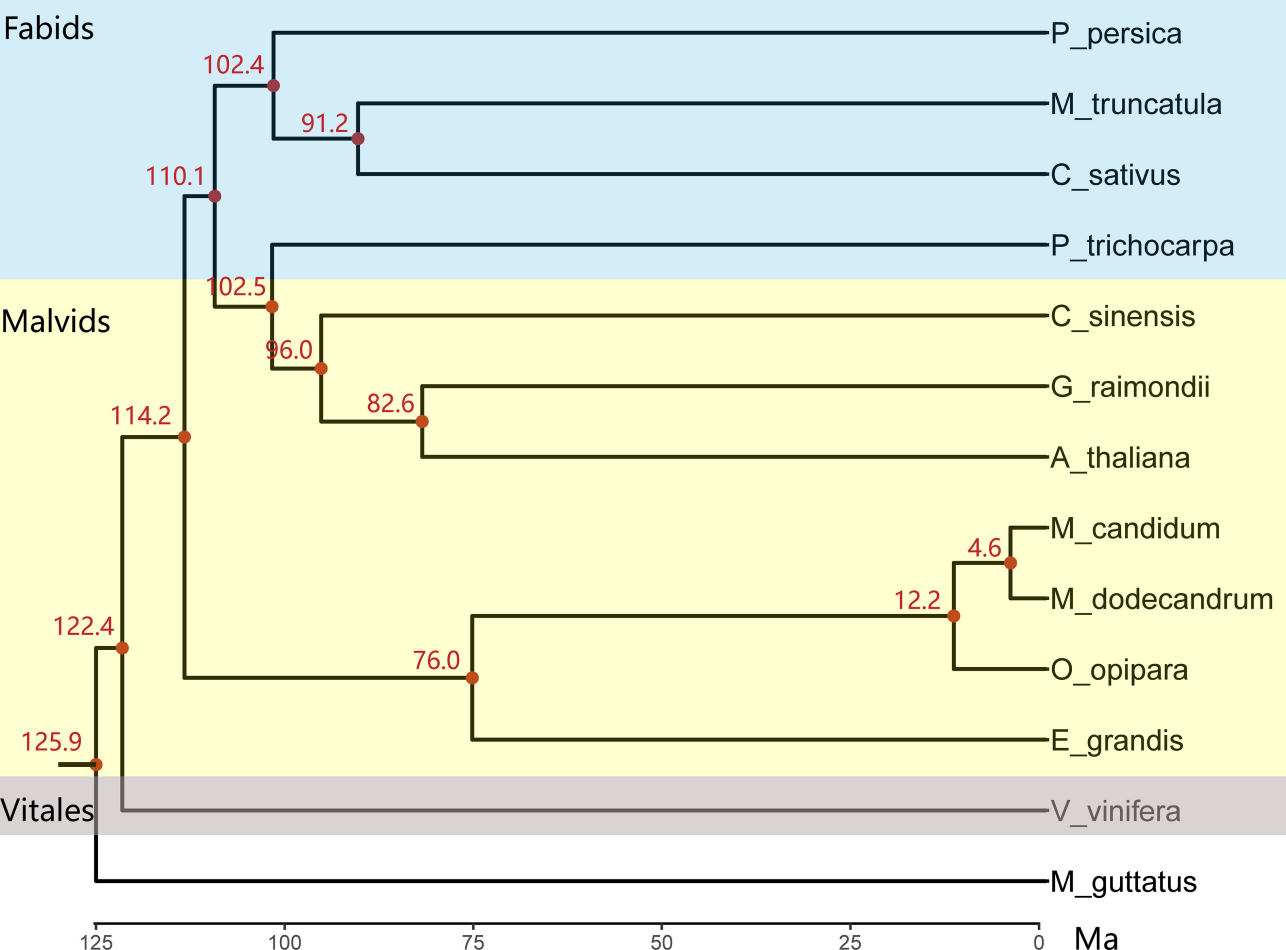
The chronogram tree of 12 rosids species and one outgroup based on the concatenated sequences of 346 single-copy genes. All nodes have a 100% bootstrap support value. The three different background colors (top to bottom) represent Fabids, Malvids and Vitales based on APG IV (2016), respectively. Species abbreviations: A_thaliana, *Arabidopsis thaliana*; C_sinensis, *Cirtus sinensis*; C_sativus, *Cucumis sativus*; E_grandis, *Eucalyptus grandis*; G_raimondii, *Gossypium raimondii*; M_truncatula, *Medicago truncatula*; M_dodecandrum, *Melastoma dodecandrum*; M_candidum, *Melastoma candidum*; O_opipara, *Osbeckia opipara*; P_trichocarpa, *Populus trichocarpa*; P_persica, *Prunus persica*; V_vinifera, *Vitis vinifera*; M_guttatus, *Mimulus guttatus*.

The topology of the constructed maximum likelihood tree of 13 species including *M. candidum* based on sequences of 346 single copy genes is consistent with previous studies (Myburg et al., 2014; Hao et al., 2022) and confirms that Myrtales is sister to the ancestor of Fabids and non-Myrtales Malvids (**Figure 3**), but is not consistent with its position shown in APGIV (2016). *Melastoma* is sister to *Osbeckia*, which is in agreement with previous studies (Veranso-Libalah et al., 2017). In the tree, the *Melastoma* and *Osbeckia* clade is then sister to *Eucalyptus*, another species with available genome from Myrtales. The ancestral branch leading to *Melastoma* and *Osbeckia* (0.306) is roughly twice as long as the *Eucalyptus* branch (0.154) (**Figure S4**), suggesting accelerating evolution for the branch leading to *Melastoma* and *Osbeckia* after diverging from *Eucalyptus*. This is likely the consequence of much shorter generation time of *Melastoma* and *Osbeckia* compared with the tree genus *Eucalyptus*. Within *Melastoma*, *M. candidum* and *M. dodecandrum* have extremely short branch length (0.005 and 0.014), indicating very recent divergence between them. Considering that *M. dodecandrum* is the first-diverging species of the genus *Melastoma*, the whole genus should have a short evolutionary history of species diversification. The divergence time between *M. candidum* and *M. dodecandrum* was dated back to 4.6 Ma (**Figure 3**), larger than the previous estimation of 1-2 Ma (Renner and Meyer, 2001). However, whether 1-2 Ma or 4.6 Ma is a fairly short evolutionary time for the formation of about 100 species in this genus, both supporting rapid speciation.

### 2.3 Genomic synteny between *M. candidum* and *M. dodecandrum*

Genomic synteny analysis between the two species shows that the 12 chromosomes are in a relatively good one-to-one correspondence between them despite the existence of some structural variations (**Figure S5**). There are 960 syntenic blocks between *M. candidum* and *M. dodecandrum*, with the number of gene pairs in these blocks ranging from 5 to 2252. A total of 57,880 gene pairs were identified in these blocks, involving 30,890 genes of *M. candidum* and 29,263 genes of *M. dodecandrum*. The inter-species syntenic blocks in the *M. candidum* genome are totally 249.3 Mb in length and contain 40,448 genes (including genes not in the gene pairs between the two species), while the counterparts in the *M. dodecandrum* genome are totally 272.6 Mb and contain 33,504 genes. The two genomes have 7.8 Mb and 13.9 Mb of non-syntenic regions, respectively. We also found that most of the syntenic blocks show a 2:2 correspondence between *M. candidum* and *M. dodecandrum* (**Figure S6**), indicating the existence of whole genome duplication in the two species.

### 2.4 Two whole genome duplications were shared by *Melastoma* and *Osbeckia*

**Figure 4:**
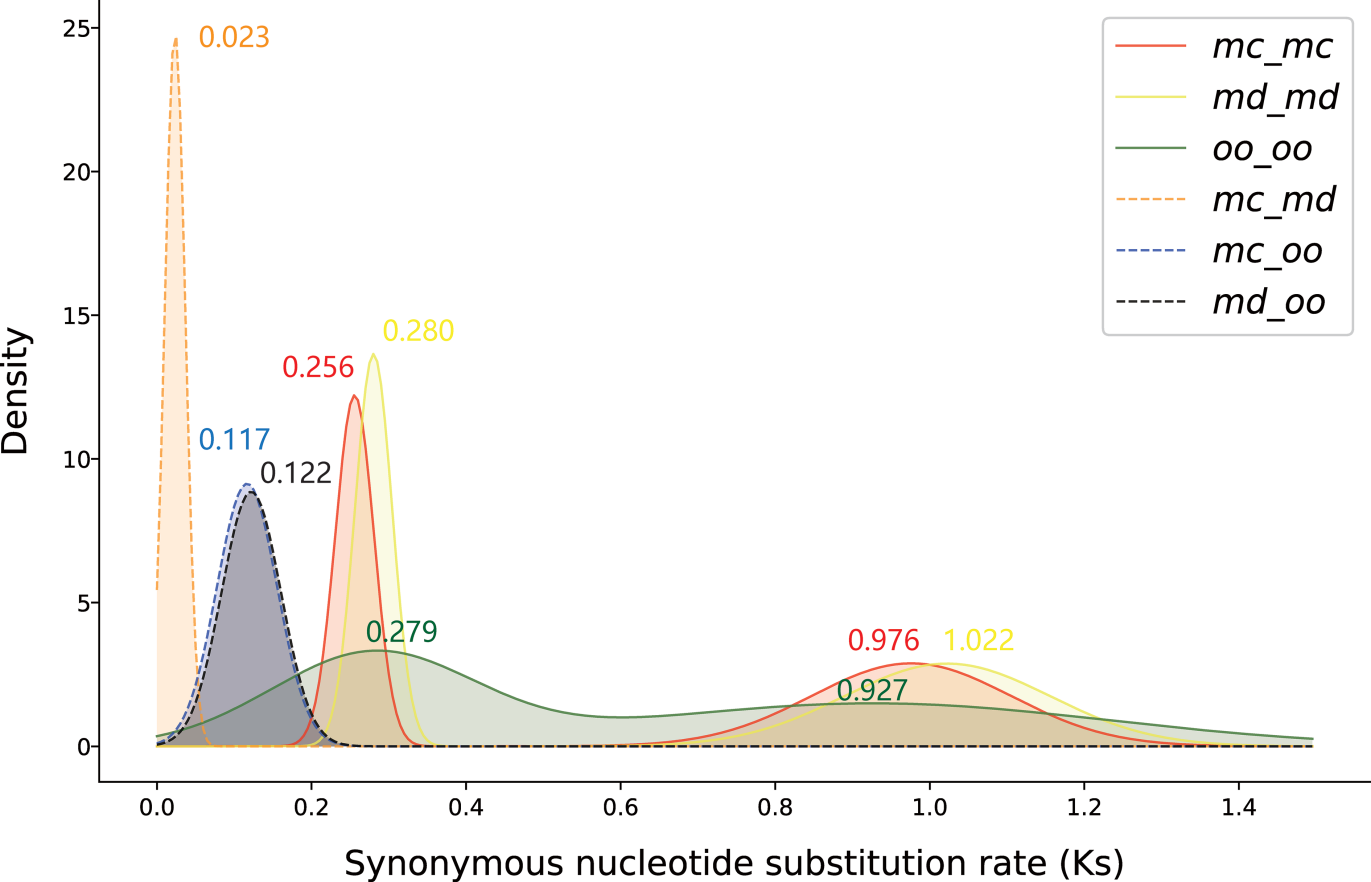
The frequency density distribution of synonymous substitution rate (Ks) of paralogous gene pairs in the syntenic blocks within, and orthologous gene pairs between, the three species, *Melastoma candidum*, *M. dodecandrum* and *Osbeckia opipara.* The values of Ks peaks are labeled. mc, *Melastoma candidum*; md, *M. dodecandrum*; oo, *Osbeckia opipara*.

1621 syntenic blocks were identified within the genome of *M. candidum*. The number of gene pairs in these blocks range from 5 to 492, with a mean of 27. The number of genes in these blocks is 27,956, covering 68.3% of the annotated genes in the genome. The synonymous substitution rate (Ks) distribution for all paralogous gene pairs in the syntenic blocks of the *M. candidum* genome have two peaks, very close to the two peaks identified in the genome of *M. dodecandrum* and the transcriptome of *Osbeckia opipara* (**Figure 4**). This implies that the two WGDs, the recent σ event at Ks = 0.256-0.280 and the more ancient ρ event at Ks = 0.927-1.022, were shared by the three species. Both of the two WGDs occurred after diverging from *Eucalyptus* (Hao et al., 2022). Because the γ triplication event is shared by all the core eudicots (Jiao et al., 2012), including *Eucalyptus* (Myburg et al., 2014), the two WGDs, both with smaller Ks peak values, must have happened after the γ event. The peaks of the Ks distribution of orthologous gene pairs between *Osbeckia opipara* and either species of *Melastoma* were much less than that for the recent WGD (**Figure 4**), further supporting the inference that the two WGDs occurred prior to the divergence of *Melastoma* and *Osbeckia*. The distribution of Ks between orthologous gene pairs in the syntenic blocks between the two species of *Melastoma* has a peak at Ks = 0.023 (**Figure 4**), again suggesting very recent divergence between them.

### 2.5 *Melastoma* specific gene families contain genes related to trichome development

Homology clustering of protein sequences of the 12 species including *M. candidum* and *M. dodecandrum* implemented in OrthoFinder2 produced 28,371 orthologous groups⁠. Of the 40,938 predicted genes in *M. candidum*, 36,924 (90.2%) were assigned to 17,108 gene families (the percentage of unassigned genes is 9.8%), in which 503 gene families comprising 1,358 predicted genes were specific to *M. candidum* (**Table S6**). There are 2,744 gene families specific to the two species of *Melastoma*, including 4,130 *M. candidum* genes and 3,549 *M. dodecandrum* genes.

The unique gene families of *M. candidum* among the 11 species (excluding *M. dodecandrum* in this analysis) were significantly enriched for 189 GO terms (**Supplementary Excel file 1**). Many of these GO terms (Category: “Biological Process”) were associated with cell differentiation, including seed trichome differentiation (GO:0090376) and seed trichome elongation (GO:0090378), and environmental resistance, including response to red or far red light (GO:0009639) and shade avoidance (GO:0009641) (**Table 2**). These enriched gene families include some transcription factors (**Table S7**), such as bHLH, HD-ZIP, and WRKY, which were known to be implicated in trichome development in *Arabidopsis* (Chalvin et al., 2020). These genes specific to *Melastoma* may contribute to trichome evolution in *Melastoma*.

**Table 2:** GO Enrichment analysis result of the gene families unique to *Melastoma*.

Category	GO term	Description	P value
Cell differentiation	GO:0090376	seed trichome differentiation	4.75e-4
	GO:0090378	seed trichome elongation	4.75e-4
	GO:0048863	stem cell differentiation	4.28e-6
	GO:0035987	endodermal cell differentiation	3.21e-4
Environment resistance	GO:0009639	response to red or far red light	2.62e-6
	GO:0009641	shade avoidance	2.37e-5
	GO:0009611	response to wounding	1.66e-4
	GO:0071236	cellular response to antibiotic	5.96e-6

### 2.6 Expanded gene families in *Melastoma* contain trichome-related transcription factors

We identified 3,027 expanded and 467 contracted gene families in *M. candidum* (**Figure S7**), among which 1,176 were significantly expanded (P < 0.05) and 287 were significantly contracted (P < 0.05). At the node of the last common ancestor leading to *M. candidum* and *M. dodecandrum*, 726 (4,120 genes) and 441 (864 genes) gene families were significantly expanded and contracted (P < 0.05), respectively. Enrichment analysis for the significantly expanded gene families in the common ancestor of *M. candidum* and *M. dodecandrum* identified some GO terms related to cell differentiation, including trichome differentiation (GO:0010026) and epithelial cell differentiation (GO:0030855), and response to environment, including defense response to fungus (GO:0050832) and response to antibiotic (GO:0046677) (**Table 3**; **Supplementary Excel file 2**). Similar to the results above, a high proportion (up to 68.6%) of genes belonging to these enriched GO terms are transcription factors, including bHLH, C2H2, HD-ZIP, WRKY, MYB, and MYB_related (**Tables 3**, **S8**).

**Table 3:** GO enrichment analysis result of significantly expanded gene families in the common ancestor of *Melastoma candidum* and *M. dodecandrum*.

Category	GO term	Description	P value	Gene number	TF %^a^	Trichome-related transcription factors (number)
Cell differentiation	GO:0010026	trichome differentiation	5.34e-3	31	48.4%	bHLH (2); C2H2 (2); HD-ZIP (3); MYB (5)
	GO:0060429	epithelium development	5.63e-6	29	20.7%	bHLH (2); C2H2 (3); HD-ZIP (1)
	GO:0030855	epithelial cell differentiation	6.05e-6	19	31.6%	bHLH (2); C2H2 (2); HD-ZIP (1)
	GO:0045595	regulation of cell differentiation	1.08e-3	44	15.9%	HD-ZIP (3); C2H2 (2); MYB (1)
	GO:0000904	cell morphogenesis involved in differentiation	8.36e-3	60	25.0%	bHLH (2); C2H2 (2); HD-ZIP (3); MYB (5)
Response to environment	GO:0050832	defense response to fungus	1.41e-8	83	42.2%	WRKY (13); MYB (12)
	GO:0002833	positive regulation of response to biotic stimulus	1.66e-8	44	34.1%	WRKY (13); MYB (2)
	GO:0032101	regulation of response to external stimulus	1.77e-3	53	28.3%	WRKY (13); MYB (2)
	GO:0031347	regulation of defense response	4.31e-3	61	24.6%	WRKY (13); MYB (2)
	GO:0046677	response to antibiotic	1.26e-7	86	68.6%	WRKY (13); MYB (35); MYB_related (4)

a The percentage of transcription factors (TF) in enriched GO terms.

Among the 18 transcription factor families with more than 10 members and fitting the normal distribution in gene number among the 12 species, 11 are largest, and 7 are the second or third largest in *M. candidum* (**Table S9**), including all six trichome-related transcription factor families shown in **Table 3**. Ten transcription factor families in *M. candidum* have a Z-score > 1.5, including HD-ZIP, MYB, WRKY, and bHLH (**Table S10**), which are also trichome-related transcription factors with enriched GO terms listed in **Table 3**. The Z-score results are consistent with the gene family evolution analysis, suggesting that *M. candidum* has more trichome-related transcription factor families than 10 other species.

### 2.7 Preferential retention of trichome-related genes following the WGDs

According to the criteria in Methods, 8,608 genes (21.0% of the total genes) have been retained from the σ event, much larger than those (3,858 genes; 9.4%) retained from the ρ event. Among the transcription factor families in *M. candidum*, including bHLH, C2H2, WRKY, HD-ZIP, MYB and MYB-related, roughly half (40.6%-50.3%) of members in these transcription factor families are retained after WGDs in *M. candidum* (**Table 4**). We identified the retention of transcription factors in the two WGDs, including 98 bHLH, 59 C2H2, 65 WRKY, 34 HD-ZIP, 93 MYB, and 30 MYB-related genes following the σ event (**Table 4**). For the ρ event, a smaller number of genes were identified, including 43 bHLH, 27 C2H2, 33 WRKY, 7 HD-ZIP, 35 MYB, and 22 MYB-related genes (**Table 4**). Whether for all the transcription factor families as a whole or for individual families, the number of genes retained after the σ event far exceeds that retained after the ρ event, which may be explained by more recent occurrence of the σ event.

**Table 4:** The number of genes in the genome and the number of genes retained after two WGDs (σ and ρ events) for six transcription factor families in *Melastoma candidum*.

	bHLH	C2H2	WRKY	HD-ZIP	MYB	MYB-related
# of genes in the genome	229	158	147	81	260	101
# of genes retained after σ+ρ events	114	71	74	35	111	41
# of genes retained after σ event	98	59	65	34	93	30
# of genes retained after ρ event	43	27	33	7	35	22

However, GO terms for trichome morphogenesis (GO:0010090) and trichome differentiation (GO:0010026) were enriched in genes retained after the ρ event, but not in genes retained after the σ event (**Table 5**; **Supplementary Excel file 3**, **4**). In addition, genes retained after the ρ event was enriched for morphogenesis of a polarized epithelium and regulation of morphogenesis of an epithelium, whereas genes retained after the σ event was enriched for epithelium development and regulation of cell differentiation (**Table 5**; **Supplementary Excel file 3**, **4**). This suggests that although more genes were retained after the σ event, trichome formation and development-related genes were mainly expanded and retained after the ρ event, and downstream genes for epithelial cell development and regulation were also retained after the σ event. The two WGD events together contributed to the retention of trichome-related genes and provided the genetic materials for trichome evolution in *Melastoma* as well as *Osbeckia*, thus facilitating their adaptation to different habitats.

**Table 5:** GO enrichment analysis result of the genes retained after two WGDs in *Melastoma candidum*.

WGD	GO term	Description	P value
σ event	GO:0045595	regulation of cell differentiation	1.77e-3
	GO:0060429	epithelium development	1.05e-3
	GO:0090558	plant epidermis development	1.27e-3
ρ event	GO:0010090	trichome morphogenesis	4.67e-3
	GO:0010026	trichome differentiation	1.60e-3
	GO:1905330	regulation of morphogenesis of an epithelium	1.22e-3
	GO:0001738	morphogenesis of a polarized epithelium	2.75e-3

## 3 Discussion

Trichomes are the product of epidermal cell differentiation (Hülskamp, 2004; Yang and Ye, 2013). Plant trichome development is coordinated and regulated by a complex network of regulatory genes, non-coding RNA, hormones, and environmental factors (Wang et al., 2019; Wang et al., 2021). In *Arabidopsis*, two acting together models, activator-depletion and activator-inhibitor models, have been well-established and proposed for explaining the molecular regulatory mechanisms of trichome development (Wang et al., 2021). Many transcription factors, including MYB-bHLH-WD40 complex, are involved and form the hub of regulating plant trichome initiation, growth and differentiation (Yang and Ye, 2013; Wang et al., 2019). The positive regulators are represented by the R2R3-MYB transcription factor GLABRA1 (GL1) and its counterparts MYB23, the bHLH transcription factor GL3 and its close homolog ENHANCER OF GLABRA3 (EGL3), and the WD40-repeat transcription factor TRANSPARENT TESTA GLABRA1 (TTG1) (Li et al., 2009b; Zhao et al., 2012). GL1/MYB23, GL3/EGL3 and TTG1 are combined to form a MYB-bHLH-TTG1 complex (Serna and Martin, 2006). This regulatory complex simulates trichome initiation by promoting the expression of GL2 and TTG2, which encode a homeodomain-leucine zipper (HD-Zip) and a WRKY transcription factor, respectively (Johnson et al., 2002). GL3-dependent depletion of TTG1 in trichome neighboring cells is the core foundation of activator-depletion model. The negative regulatory factors mainly consist of genes encoding single-repeat R3 MYB proteins, including CAPRICE (CPC), TRIPTYCHON (TRY) (Szymanski et al., 2000). They combine with GL3/EGL3 and TTG1 by competing with GL1/MYB23 to form an inactivating complex, thereby inhibiting trichome formation (Esch et al., 2003; Yang et al., 2020). ENHANCER OF TRY AND CPC 1 (ETC1) and ETC2 act as enhancers of TRY and CPC. Activator-inhibitor model is explained by TRY/CPC to form an inactive TRY/CPC-GL3/EGL3-TTG1 complex, which negatively regulates trichome formation by replacing the transcription factor GL1/MYB23. These transcription factors form the hub of regulating trichome initiation and differentiation. In addition, cytokinin (CK) increases trichome formation through C2H2 transcription factors (Wang et al., 2021).

The trichome of *Arabidopsis* has been intensively studied as a model for cell differentiation, but trichome development in different plants and organs can be regulated by different mechanisms. For example, homologs of GL1, GL2, TTG1, and HD-ZIP in cotton were reported to have similar function to those in *Arabidopsis*, but the negative regulators like single-repeat R3 MYB transcription factors have not been identified in cotton (Zhang et al., 2010; Yang and Ye, 2013). It is unknown whether the activator-inhibitor model is effective in cotton fiber (trichome on its seed) development. Also, the two types of multicellular trichomes, short- and long-stalked, produced in tobacco are explained by different developmental mechanisms (Payne et al., 1999). The multicellular trichomes including those of *Melastoma* may be controlled by mechanisms that are more complex than those of unicellular trichomes such as *Arabidopsis* and cotton. *Melastoma* has the advantage to dissect the mechanisms because a high level of trichome diversity is displayed among species of this genus.

The high-quality genome assembly of *M. candidum* makes it feasible to provide genomic insights into the evolution of trichomes, a key trait in contributing to species diversification and ecological adaptation in *Melastoma* (Wong, 2016; Ng et al., 2019; Huang et al. unpublished data). Based on the remarkable trichome diversity in *Melastoma*, we predict the expansion of trichome-related genes in the *M. candidum* genome, which can provide the raw materials for trichome evolution and thus effective response to diverse biotic and abiotic stresses. Our genomic analysis results are consistent with this prediction.

First, GO enrichment analysis of gene families specific to, and significantly expanded in *Melastoma* found that both were enriched for GO terms related to cellular differentiation (including trichome differentiation) and environmental response. This involves six transcription factor families, including C2H2, bHLH, HD-ZIP, WRKY, MYB, and MYB-related, which are key regulators of trichome formation and differentiation in plants such as *Arabidopsis* (Wang et al., 2019; Wang et al., 2021), as detailed below. Large and frequent changes in gene family size among species might be associated with some important morphological, physiological, and behavioral differences among them (Demuth and Hahn, 2009).

Meanwhile, we provided genomic evidence that two WGDs (the σ and ρ events) happened in, and shared by *Melastoma* and *Osbeckia*. After a WGD event, one of the duplicated genes may be lost or become pseudogenes or both duplicates may be retained *via* sub-functionalization or neo-functionalization (Edger and Pires, 2009; Li et al., 2016; Li et al., 2021). Given that both *Melastoma* and *Osbeckia* have a great variety of trichomes, we propose the hypothesis that the two WGDs allowed the expansion and retention of trichome-related genes. Our enrichment analysis of genes duplicated and retained by the two WGDs found that the two WGD events contributed to preferential retention of many trichome-related transcription factors, and GO terms including trichome morphogenesis and trichome differentiation were enriched after the ρ event. This supports our hypothesis that the ρ event allowed the expansion and retention of genes promoting trichome development, and the event σ further duplicated and retained trichome-associated genes. Therefore, both WGD events have contributed to retention of trichome-related genes.

In summary, trichome-associated transcription factors were identified in *Melastoma*-specific, significantly expanded, and preferentially retained genes after two WGDs. These transcription factors have been shown to be central components of the regulatory network for trichome formation and differentiation in model plants such as *Arabidopsis* and cotton. Therefore, we suggest that these expanded genes, especially duplicated and retained transcription factors in the ρ event, provide the raw genetic materials for trichome evolution and further contribute to ecological adaptation of *Melastoma*.

## 4 Conclusion

We assembled and annotated a high-quality, chromosome-level genome of *M. candidum*. Genomic data support very recent divergence between *M. candidum* and *M. dodecandrum* (Ks peak of the orthologous gene pairs at 0.02) and good synteny of 12 chromosomes between them. Two WGD events were identified in, and shared by *Melastoma* and *Osbeckia*, two sister genera both with a high level of trichome diversity. We found that the gene families involved in trichome initiation and differentiation were significantly expanded, and meanwhile, trichome-related genes, especially related transcription factor genes, were preferentially retained following the two WGDs, which together may greatly contribute to trichome evolution in *Melastoma*. Since trichomes in species of *Melastoma* contribute to their adaptation to diverse environments, the expansion and retention of trichome-related genes may promote rapid species diversification in this genus. The *Melastoma* genome also provides an ideal genomic resource for ecological and evolutionary studies in this genus, particularly transcription factor genes in association with trichome evolution.

## 5 Materials and methods

### 5.1 Plant materials and sequencing

One individual of *Melastoma candidum* from Wenchang, Hainan, China was collected and transplanted in the campus of Sun Yat-sen University (SYSU) and used for *de novo* genome sequencing. Total DNA was extracted from fresh leaves of this individual using the modified cetyltrimethylammonium bromide (CTAB) protocol (Doyle, 1991)⁠. RNA was isolated from leaves, flowers, young branches, fruits and two whorls of stamens of the individual using the method described in (Fu et al., 2004).

Four DNA libraries with the insert sizes of 180 bp, 300 bp, 500 bp and 800 bp were constructed and then sequenced on an Illumina Hiseq2000 platform. A PacBio library with an insertion size 20 Kb was also constructed and sequenced on the PacBio RSII sequencer with DNA Sequencing Kit 2.0 (Pacific Biosciences, CA, USA) (**Table S11**). Transcriptome libraries using RNA isolated from the six tissues mentioned above were constructed and then sequenced separately on an Illumina Hiseq2000 platform. Moreover, a Hi-C library following a standard procedure (Lieberman-Aiden et al., 2009) was constructed, and then sequenced on an Illumina HiSeq X Ten sequencer. All the details of these sequencing data were shown in **Table S11**. In addition, the fresh leaves of one individual of *Osbeckia opipara* sampled from Chishui, Guizhou, China was used for transcriptome sequencing using the same method for *Melastoma*.

### 5.2 Genome size estimation

Illumina reads were filtered by fastp 0.20.1 (Chen et al., 2018) and FastUniq (Xu et al., 2012) with default parameters. All clean reads were supplied to Jellyfish v.2.3.0 (Marçais and Kingsford, 2011)⁠ to calculate Kmer (k = 21) frequency. The genome size, as well as the heterozygosity and repeat content were then estimated in GenomeScope 1.0 (Vurture et al., 2017).

### 5.3 Genome assembly, annotation and quality assessment

A two-step procedure was implemented to assemble the draft genome of *M. candidum.* First, *de novo* assembly was implemented using ALLPATH-LG v52488 (Gnerre et al., 2011) with default settings except for the two parameters: ploidy set to 2, and estimated genome size set to 257 Mb. At this stage, only Illumina reads were supplied into the assembler, and corrected with the embedded modules PreCorrect and FindErrors with 24-kmer read stacks. Next, the pre-assembled contigs of *M. candidium* were scaffolded by SSPACE v3.0 (Boetzer et al., 2011), and the gaps were closed with Gapclose v1.12 (Luo et al., 2012). The PacBio subreads were corrected with LoRDEC v0.9 (Salmela and Rivals, 2014) and were then used to fill gaps and scaffolding all the available scaffolds with PBJelly v14.1.15 (Patel and Jain, 2012).

After mapping the clean Hi-C reads against the scaffolds using BWA v0.7.12-r1039 (Li and Durbin, 2009) with default parameters, we corrected, clustered, sorted, and anchored the scaffolds >1 kb into 12 pseudomolecules using Juicer v1.6 (Durand et al., 2016) and 3D-DNA (Dudchenko et al., 2017). Then, Juicebox Assembly Tools (https://github.com/aidenlab/Juicebox) was used to manually review the scaffolds and plot the contact maps. The final genome of 12 pseudochromosomes was obtained with the run-asm-pipeline-post-review.sh script in 3D-DNA. Finally, we clipped scaffolds < 1 kb in length.

EDTA v1.9.6 (Ou et al., 2019) was used to identify repetitive sequences with default parameters. Noncoding RNA including rRNA, tRNA, miRNA, snoRNA were predicted using INFERNAL v 1.1.4 (Nawrocki and Eddy, 2013) by searching the *M. candidum* genome against the RNA family database release 14.7 (RFAM v 14.7) with the parameters “-Z 512 –cut_ga –rfam –nohmmonly –fmt 2” (Kalvari et al., 2021).

Protein-coding genes were predicted using a combination of homologous-sequence search, *ab initio* gene prediction, and transcriptome-based prediction implemented in a genome annotation tool GETA v 2.4.5 (https://github.com/chenlianfu/geta). Illumina RNA-seq reads from different tissues were mapped to the genome assembly using HISAT2 (Kim et al., 2019) and were used for transcriptome-based prediction. Protein sequences from six eudicots (*Arabidopsis thaliana*, *Cirtus sinensis*, *Gossypium raimondii*, *Medicago truncatula*, *Populus trichocarpa*, and *Vitis vinifera*) and plant protein sequences from UniProtKB/Swiss-Prot (https://www.uniprot.org/) were used for homology-based prediction with GeneWise (https://www.ebi.ac.uk/~birney/wise2/). *ab initio* prediction was performed in Augustus v3.3.3 (Stanke and Morgenstern, 2005), trained with intron and exon information generated above. These prediction results were integrated and then were searched against the Pfam database for screening to get the final gene prediction result. Functional annotation of genes was performed with InterproScan (Jones et al., 2014), eggnog-mapper (http://eggnog-mapper.embl.de/), PANNZER2 (Törönen et al., 2018), and Mercator4 v3.0 (Schwacke et al., 2019).

We used four approaches to assess the quality of genome assembly and annotation of *M. candidum*. First, genome continuity and completeness were assessed using QUAST v5.1.0 (Gurevich et al., 2013) to count the scaffold N50, L50, N90 and L90 of the genome. Second, Illumina DNA reads, PacBio reads and RNA-seq reads were mapped to the genome using BWA-MEM (Li, 2013), Minimap2 (Li, 2018) and HISAT2 v2.1.0 (Kim et al., 2019), respectively. The accuracy of the genome was assessed by the analysis of sequencing depth, percentage of mapped reads and genome coverage using SAMtools (Li et al., 2009a), bamdst (https://github.com/shiquan/bamdst) and Qualimap 2 (Okonechnikov et al., 2015). Third, the completeness of genome assembly and annotation was assessed using BUSCO v5.1.3 (Simão et al., 2015) with both eudicots_odb10 and embryophyta_odb10 databases. The *M. dodecandrum* genome published before (Hao et al., 2022) was also assessed with the same method. Finally, the continuity of the genome was also assessed by LTR Assembly Index (LAI) using LTR_retriever v2.9.0 (Ou et al., 2018; Ou and Jiang, 2018).

### 5.4 Transcriptome assembly and assessment of *Osbeckia opipara*

Illumina reads of *Osbeckia opipara* were first trimmed for quality using fastp 0.20.1 (Chen et al., 2018). The clean reads were *de novo* assembled to 159,724 transcripts using Trinity v2.11.0 (Haas et al., 2013) with default parameters. The transcripts were clustered using cd-hit-est v4.8.1 (Li and Godzik, 2006)with the identity parameter set to 0.95% and the longest transcript for each cluster was selected using the script get_longest_isoform_seq_per_trinity_gene.pl in Trinity, which led to the output of 64,927 unigenes. The BUSCO assessment of these unigenes revealed 84.9% and 89.7% of the complete BUSCOs in the eudicots_odb10 and embryophyta_odb10 dataset, respectively. TransDecoder v5.5.0 was employed to predict coding regions of these unigenes and then to translate them into amino acid sequences. Only sequences > 150 aa in length were kept for subsequent analyses.

### 5.5 Phylogeny construction and divergence time estimation

Using OrthoFinder2 (Emms and Kelly, 2019), gene families of *M. candidum* and other 12 related species, namely, *M. dodecandrum*, *Arabidopsis thaliana*, *Cirtus sinensis*, *Cucumis sativus*, *Euclyptus grandis*, *Gossypium raimondii*, *Medicago truncatula*, *Mimulus guttatus*, *Osbeckia opipara Populus trichocarpa*, *Prunus persica*, and *Vitis vinifera* (**Table S12**), were clustered with default parameters. One-to-one orthogroups among *M. candidum* and other 12 species were identified as single copy genes. For each single copy gene, protein sequences of these species were aligned using MAFFT v7.0 (Katoh and Standley, 2013), and then their corresponding nucleotide sequences were aligned using the pal2nal.pl script (Suyama et al., 2006). All the nucleotide sequence alignments were concatenated into a supermatrix, and then subject to substitution model test using ModelFinder (Kalyaanamoorthy et al., 2017) with the Bayesian information criterion (BIC). The maximum-likelihood tree between *M. candidum* and other 12 species was constructed under GTR+F+R4 model using IQ-TREE v2.0.3 (Nguyen et al., 2014) with 1000 ultrafast bootstrap replicates (Hoang et al., 2017) and *Mimulus guttatus* as an outgroup.

The divergence time in the ML tree was estimated by mcmctree program in the PAML package (Yang, 2007) under a relaxed clock model with independent rates constraints and two calibration points, one between Asterids and Rosids (111-131 million years ago, Ma), and the other between *Arabidopsis* and *Populus* (98-117 Ma) from TimeTree (http://www.timetree.org).

### 5.6 Enrichment analysis of *Melastoma* specific gene families

For this analysis, we redid the gene family clustering for the 12 species in OrthoFinder2 by excluding *Osbeckia opipara* because it has only transcriptome data and genes are not adequate to represent those in its genome. We extracted the gene families specific to *Melastoma* by combining gene families both specific to the two species of *Melastoma* and specific to *M. candidum*. Enrichment levels of Gene Ontology (GO) terms were evaluated by comparing genes in the *Melastoma* specific gene families with the genomic background (all annotated genes of *M. candidum*) in the clusterProfiler v4.2.2 package (Wu et al., 2021) of R. Statistical significance was tested by Fisher’s exact test (Fisher, 1922) and adjusted *P* values were calculated according to the Benjamini and Hochberg (false discovery rate) method (Benjamini and Hochberg, 1995). We used default parameters except for pAdjustMethod = “BH”, pvalueCutoff = 0.05, and qvalueCutoff = 0.2.

### 5.7 Gene family expansion and contraction analysis

Gene family clustering results for the 12 species in the preceding section were used for this analysis. We analyzed changes in gene family size across a specified chronogram tree of the 12 species using 601 single copy genes of these species in CAFÉ 5 (Mendes et al., 2020). Gene gain and loss rates were modeled using a birth and death process. Poisson distribution was specified as root frequency distribution model. Gene families >100 members were filtered with the script clade_and_size_filter.py. Evolutionary rates were estimated using different K values (evolutionary rate categories) ranging from 2 to 8. Birth and death rate with the maximum likelihood value (K = 4) were used to infer ancestral states of gene family sizes for each node and changes along each branch in the phylogenetic tree. Gene families with significant expansion and contraction were determined with a threshold conditional P-value (P < 0.05). Changes of gene family size along each branch were labeled in the phylogenetic tree. GO enrichment analysis of the significantly expanded gene families in the common ancestor of the two species of *Melastoma* was performed using the methods and parameters mentioned above.

### 5.8 Genomic synteny analysis and whole genome duplication identification

All-versus-all alignment of the protein sequences of *M. candidum* was constructed using the blastp algorithm (Altschul et al., 1997). To detect the signature of whole genome duplication (WGD), the icl module in WGDI v0.5.1 (Sun et al., 2022) was employed to define syntenic blocks with minimum gene number of five and evalue threshold of 1e-5 in the blast search. For each gene pair in the syntenic blocks, synonymous nucleotide substitution rate (Ks) was calculated by the ks module in WGDI with the YN00 model. Tandems and blocks with significance more than 0.1 were filtered and by the kp module with parameters “tandem = true; pvalue = 0.1”. To avoid random errors and the effect of synonymous substitution saturation, we retained gene pairs with the Ks values > 0.05 and Ks values < 1.50, which is the upper limit of the divergence between *Melastoma* and *Eucalyptus* (Hao et al., 2022). According to color of dotplots and troughs value (0.6) of Ks frequency distribution, syntenic blocks from the older and younger WGDs were separated by the kp module with parameters “homo = 0,0.5; ks_area = 0.6,1.5” and “homo = 0.5,1; ks_area = 0.05,0.6”, respectively. The frequency distribution of Ks for each of the WGDs was fitted individually a normal distribution with Gaussian model using the pf module with “mode=median”, and then the kf module was used to make a plot based on the fitted parameters. Intra-genomic syntenic blocks of *M. dodecandrum* were analyzed with the same method.

For the *O. opipara* transcriptome, the gene families were constructed using the mclblastline pipeline (Enright et al., 2002), and each gene family was compared using MUSCLE (Edgar, 2004), and finally the codeml module in the PAML package (Yang, 2007) was used to calculate the Ks values with the YN00 model. The script KSPloter.py (https://github.com/EndymionCooper/KSPlotting) was employed to execute the above process with mode 1 (-R M1).

Meanwhile, we extracted single copy genes of *M. candidum*, *M. dodecandrum* and *O. opipara* as a representative of orthologs to calculate pairwise Ks of gene pairs among the three species. For each gene pair, the protein sequences were aligned and then converted into coding sequence alignments by ParaAT (Zhang et al., 2012). The Ks value was calculated using KaKs_Calculator 2.0 (Wang et al., 2010) with the YN00 model. Gene pairs with the Ks values > 0.05 and < 1.50 were retained. The frequency distribution of Ks for each peak is constructed with 200 bins and is fitted a normal distribution with a Gaussian model.

The density of genes, repeats, genes within the syntenic blocks, and GC content in the 12 pseudochromosomes of *M. candidum* were calculated in a 100-kb sliding window with BEDTools v2.30.0 (Quinlan and Hall, 2010) and were plotted with syntenic curves between chromosomes using Circos v 0.69-8 (Krzywinski, 2009). Syntenic regions between the 12 pseudochromosomes of *M. candidum* and *M. dodecandrum* were identified and plotted using the MCScan pipeline (Tang et al., 2008).

### 5.9 Gene retention analysis after the WGDs

Based on the phylogenetic tree constructed above, gene duplication events were identified with parameters “-M msa -T raxml” in OrthoFinder2. We firstly conducted gene trees for each orthogroup using maximum likelihood method. Then, reconciliation of all the nodes of gene trees with corresponding nodes in the species tree was executed to obtain resolved gene trees and to further infer gene duplication events.

Firstly, orthogroups containing four and more genes and at least one gene from non-*Melastoma* species were kept. Then, we extracted gene duplication events specific to *Melastoma* and screened the gene family trees to accurately identify gene duplication events with the two criteria: 1) Both of the two child branches of each gene duplication event have genes from *M. candidum*, and 2) The bootstrap support values are not less than 0.5. We further eliminated tandem duplications when two duplicated genes located within the range of five genes. Finally, we got 1,026 gene duplication events.

Pairwise protein sequences for all duplicated genes were aligned and then converted into nucleotide sequence alignments using ParaAT. Ks value for pairwise comparisons at the duplication node (one gene in one child branch and the other gene in another child branch) were calculated using the YN00 model implemented in KaKa_Calculator2.0. The mean of Ks for each gene duplication event were then calculated. We filtered out gene duplication events with Ks mean value < 0.05 and > 1.50. To further validate if the duplicate genes are still located on syntenic blocks, we extracted duplicate genes in the syntenic blocks based on the WGD analysis results. To distinguish genes duplicated by the two WGDs, we defined that duplicated gene pairs in the syntenic blocks produced by the σ event belong to the retained genes after the σ event and so do gene pairs that were produced by the ρ event. GO enrichment analysis of the retained genes after the two WGDs was performed using the same methods mentioned above.

### 5.10 Transcription factors retention analysis

The transcription factor families for the two species of *Melastoma* were annotated PlantTFDB v5.0 (Jin et al., 2016; Tian et al., 2019), and transcription factors of 10 other species were download from PlantTFDB v5.0. We examined whether the gene number of each family from the 12 species fits a normal distribution using the Shapiro-Wilk Test. Only transcription factor families with a normally distributed gene number across the 12 species were retained, and those with very few members (< 10) in any species were removed. For each screened family, we calculated the z-score according to the formula z = (x-μ)/σ, in which x, μ and σ represent the gene number of the family in *M. candidum*, the mean and the standard deviation of gene numbers of this family in the 12 species, respectively. We then analyzed the retention of transcription factor genes after the two WGDs. Using the same method as the gene duplication retention analysis described above, we identified the retained transcription factor genes of *M. candidum* for each of the two WGDs.

## Data availability statement

All raw reads of *Melastoma candidum*, including the Pacbio, Hi-C, Illumina DNA-seq, and RNA-sequencing, were deposited in the National Center for Biotechnology and Information (NCBI) short read archive repository under the accession numbers SRR22574044-SRR22574048. The genome assembly and annotation of *Melastoma candidum* was deposited in NCBI GenBank under the accession number: JAKZET000000000. (BioProject accession: PRJNA811312). The RNA-sequencing raw reads of *Osbeckia opipara* was deposited in the NCBI short read archive repository under the accession number SRR22557471. The *de novo* assembly was deposited in NCBI Transcriptome Shotgun Assembly Sequence Database under the accession number: GKED00000000. (BioProject accession: PRJNA909408).

## Author contributions

RZ and SD planned the projects. YZ analyzed data and wrote the manuscript. CS and PZ performed the experiments. WW and YL participated in plant sampling and sequencing. WW participated in data analysis, and YZ and RZ revised the manuscript. All authors contributed to the article and approved the submitted version.

## Footnotes

1 Huang G., Wu W., Chen Y., Zhi X., Zou P., Ning Z., Fan Q., Liu Y., Deng D., Zeng K., Zhou R. Balancing selection on an MYB transcription factor maintains the twig trichome color variation in Melastoma normale. unpublished.
