# Supplementary material for: Chromosomal-level genome assembly of Melastoma candidum provides insights into trichome evolution
Source: Front Plant Sci. 2023 Jan 27;14:1126319. doi: 10.3389/fpls.2023.1126319 (PMC9911893; doi:10.3389/fpls.2023.1126319)
Supplement: Supplementary file 1 [file DataSheet_1.docx]

# Supplementary Tables

### Table S1. Read mapping rates and coverage of the *Melastoma candidum* genome

| Sequencing platform | Library type | Mapping rate | Genome coverage |
| --- | --- | --- | --- |
| Illumina | DNA-seq | 97.94% | 99.58% |
| PacBio | DNA-seq | 96.02% | 99.84% |
| Illumina | RNA-seq (fruit) | 97.22% | - |
| Illumina | RNA-seq (flower) | 97.19% | - |
| Illumina | RNA-seq (leaf) | 87.00% | - |
| Illumina | RNA-seq (branch) | 95.10% | - |
| Illumina | RNA-seq (stamen-outer whorl) | 97.56% | - |
| Illumina | RNA-seq (stamen-inner whorl) | 96.88% | - |

### Table S2. BUSCO assessment for the *Melastoma candidum* genome assembly with the eudicots_odb10 and embryophyta_odb10 datasets

| Class | eudicots_odb10 | | embryophyta_odb10 | |
| --- | --- | --- | --- | --- |
|  | Number of genes | Percentage | Number of genes | Percentage |
| Complete BUSCOs | 2162 | 92.9% | 1558 | 96.6% |
| Complete and single-copy BUSCOs | 1685 | 72.4% | 1260 | 78.1% |
| Complete and duplicated BUSCOs | 477 | 20.5% | 298 | 18.5% |
| Fragmented BUSCOs | 41 | 1.8% | 21 | 1.3% |
| Missing BUSCOs | 123 | 5.3% | 35 | 2.1% |
| Total BUSCOs | 2326 | - | 1614 | - |

### Table S3. Summary of repetitive sequences in the *Melastoma candidum* genome

| Repeat Class | Number | Length(bp) | % in the genome |
| --- | --- | --- | --- |
| LTRs |  |  |  |
| Gypsy | 24731 | 29861272 | 11.66 |
| Copia | 18459 | 20020279 | 7.82 |
| unknown | 15574 | 9269027 | 3.62 |
| TIRs |  |  |  |
| CACTA | 3579 | 1428726 | 0.56 |
| Mutator | 25930 | 10061594 | 3.93 |
| PIF/Harbinger | 883 | 306067 | 0.12 |
| Tc1/Mariner | 256 | 117004 | 0.05 |
| hAT | 4716 | 1996262 | 0.78 |
| nonTIR |  |  |  |
| helitron | 5282 | 2075612 | 0.81 |
| repeat_region |  |  |  |
|  | 18678 | 5491770 | 2.14 |
| Total | 118088 | 80627613 | 31.49 |

### Table S4. Functional annotation of protein-coding genes of *Melastoma candidum*

| **Database** | **Number of genes annotated** | **% of total genes** |
| --- | --- | --- |
| Total | 37,393 | 91.34 |
| Interproscan | 33,144 | 80.96 |
| Eggnog | 35,777 | 87.39 |
| Pannzer2 | 29,218 | 71.37 |
| Mercator4 | 32,469 | 79.31 |

### Table S5. BUSCO assessment for the *Melastoma candidum* proteome with the eudicots_odb10 and embryophyta_odb10 datasets

| Class | eudicots_odb10 | | embryophyta_odb10 | |
| --- | --- | --- | --- | --- |
|  | Number of genes | Percentage | Number of genes | Percentage |
| Complete BUSCOs | 2175 | 93.5% | 1550 | 96.0% |
| Complete and single-copy BUSCOs | 1574 | 67.7% | 1170 | 72.5% |
| Complete and duplicated BUSCOs | 601 | 25.8% | 380 | 23.5% |
| Fragmented BUSCOs | 24 | 1.0% | 18 | 1.1% |
| Missing BUSCOs | 127 | 5.5% | 46 | 2.9% |
| Total BUSCOs | 2326 | - | 1614 | - |

### Table S6. Summary of gene family clustering of *Melastoma candidum* and 11 other species

| **Species** | Number of genes | Number of genes in orthogroups | Percentage of genes in orthogroups | Number of species-specific orthogroups | Number of genes in species-specific orthogroups |
| --- | --- | --- | --- | --- | --- |
| *Arabidopsis thaliana* | 27416 | 25088 | 91.5 | 737 | 3494 |
| *Cirtus sinensis* | 25379 | 23669 | 93.3 | 274 | 901 |
| *Cucumis sativus* | 21503 | 19674 | 91.5 | 150 | 548 |
| *Eucalyptus grandis* | 36349 | 31273 | 86 | 783 | 3700 |
| *Gossypium raimondii* | 37505 | 34716 | 92.6 | 670 | 2776 |
| *Medicago truncatula* | 50894 | 44294 | 87 | 2132 | 13515 |
| *Melastoma candidum* | 40938 | 36924 | 90.2 | 503 | 1358 |
| *Melastoma dodecandrum* | 35681 | 31393 | 88 | 526 | 1302 |
| *Mimulus guttatus* | 28140 | 26001 | 92.4 | 608 | 2954 |
| *Populus trichocarpa* | 34699 | 32243 | 92.9 | 466 | 1832 |
| *Prunus persica* | 26873 | 24475 | 91.1 | 438 | 1749 |
| *Vitis vinifera* | 31845 | 27548 | 86.5 | 800 | 2845 |

### Table S7. Transcription factor genes from *Melastoma*-specific gene families

| Gene ID | transcription factors |
| --- | --- |
| mc23540 | HD-ZIP |
| mc38966 | HD-ZIP |
| mc03015 | WRKY |
| mc09092 | WRKY |
| mc09725 | WRKY |
| mc23970 | WRKY |
| mc29284 | WRKY |
| mc29844 | WRKY |
| mc30053 | WRKY |
| mc30056 | WRKY |
| mc30525 | WRKY |
| mc00192 | bHLH |
| mc12104 | bHLH |
| mc12490 | bHLH |
| mc12538 | bHLH |
| mc21805 | bHLH |
| mc21881 | bHLH |
| mc22546 | bHLH |
| mc26251 | bHLH |
| mc32871 | bHLH |
| mc38342 | bHLH |
| mc00423 | bZIP |
| mc34126 | bZIP |

### Table S8. Transcription factor genes in the significantly expanded gene families of the last common ancestor of *Melastoma candidum* and *M. dodecandrum*

| Transcription factors | Gene IDs |
| --- | --- |
| bHLH | mc16517, mc39189 |
| bZIP | mc01386, mc02741, mc08849, mc15940, mc20150, mc24112, mc34011 |
| C2H2 | mc16197, mc17673, mc19564, mc35417, mc35647 |
| HD-ZIP | mc21106, mc29313, mc25833, mc33439 |
| WRKY | mc08820, mc11074, mc24131, mc26587, mc26588, mc32625, mc32626, mc32627, mc32628, mc32629, mc32630, mc37009, mc38247 |
| MYB | mc00355, mc01985, mc03646, mc05001, mc07029, mc07376, mc07444, mc08068, mc09483, mc09744, mc12623, mc12958, mc13007, mc13022, mc13522, mc14330, mc14688, mc17144, mc18896, mc19584, mc21961, mc22322, mc22457, mc24356, mc24684, mc24727, mc25290, mc25433, mc25964, mc25972, mc28577, mc31418, mc33277, mc33736, mc34095, mc34646, mc35439, mc37980, mc40217, mc40762 |
| MYB_related | mc03569, mc05687, mc09662, mc17378 |

### Table S9. The gene count of transcription factor families in *Melastoma candidum* and 11 other plant species

| TF | *Arabidopsis thaliana* | *Cucumis sativus* | *Cirtus sinensis* | *Eucalyptus grandis* | *Gossypium raimondii* | *Melastoma candidum* | *Melastoma dodecandrum* | *Mimulus guttatus* | *Medicago truncatula* | *Prunus persica* | *Populus trichocarpa* | *Vitis vinifera* | P value (Shapiro-Wilk Test) |
| --- | --- | --- | --- | --- | --- | --- | --- | --- | --- | --- | --- | --- | --- |
| AP2 | 18 | 18 | 16 | 22 | 32 | 30 | 23 | 26 | 27 | 19 | 31 | 19 | 0.2297 |
| ARF | 22 | 16 | 17 | 17 | 36 | 44 | 31 | 21 | 41 | 17 | 35 | 17 | 0.0253* |
| ARR-B | 14 | 15 | 10 | 7 | 20 | 21 | 12 | 14 | 29 | 9 | 17 | 12 | 0.4011 |
| B3 | 66 | 32 | 43 | 86 | 80 | 58 | 48 | 46 | 82 | 69 | 110 | 29 | 0.8262 |
| BBR-BPC | 7 | 4 | 3 | 5 | 8 | 9 | 7 | 6 | 2 | 3 | 16 | 5 | 0.0503 |
| BES1 | 8 | 6 | 7 | 6 | 11 | 15 | 12 | 9 | 7 | 7 | 14 | 6 | 0.0369* |
| C2H2 | 100 | 89 | 79 | 105 | 160 | 158 | 145 | 100 | 104 | 82 | 142 | 64 | 0.1934 |
| C3H | 50 | 37 | 41 | 40 | 75 | 70 | 64 | 44 | 55 | 50 | 62 | 43 | 0.3436 |
| CAMTA | 6 | 4 | 6 | 6 | 11 | 9 | 6 | 5 | 8 | 5 | 7 | 4 | 0.1843 |
| CO-like | 17 | 12 | 10 | 8 | 21 | 17 | 13 | 11 | 10 | 9 | 17 | 6 | 0.5398 |
| CPP | 8 | 4 | 6 | 5 | 11 | 12 | 12 | 10 | 8 | 6 | 12 | 6 | 0.1192 |
| DBB | 11 | 8 | 10 | 8 | 20 | 12 | 11 | 11 | 9 | 8 | 19 | 7 | 0.0081* |
| Dof | 36 | 36 | 24 | 26 | 60 | 68 | 68 | 28 | 42 | 25 | 45 | 22 | 0.0532 |
| E2F/DP | 8 | 7 | 7 | 5 | 10 | 9 | 6 | 10 | 6 | 7 | 9 | 7 | 0.3622 |
| EIL | 6 | 4 | 5 | 4 | 11 | 9 | 6 | 8 | 13 | 5 | 7 | 2 | 0.7014 |
| ERF | 123 | 120 | 103 | 129 | 230 | 224 | 191 | 119 | 185 | 102 | 175 | 80 | 0.2098 |
| FAR1 | 17 | 21 | 39 | 39 | 35 | 12 | 23 | 32 | 77 | 57 | 51 | 18 | 0.3363 |
| G2-like | 42 | 38 | 37 | 40 | 71 | 91 | 78 | 43 | 46 | 37 | 66 | 40 | 0.0079* |
| GATA | 30 | 25 | 22 | 23 | 46 | 46 | 46 | 26 | 43 | 20 | 39 | 19 | 0.0311* |
| GRAS | 34 | 37 | 45 | 85 | 82 | 140 | 117 | 41 | 67 | 48 | 106 | 43 | 0.0945 |
| GRF | 9 | 8 | 8 | 6 | 18 | 19 | 21 | 9 | 8 | 10 | 19 | 8 | 0.0058* |
| GeBP | 22 | 5 | 5 | 2 | 8 | 10 | 11 | 9 | 6 | 8 | 6 | 1 | 0.0418* |
| HB-PHD | 2 | 2 | 2 | 1 | 4 | 4 | 5 | 2 | 2 | 2 | 4 | 2 | 0.0084* |
| HB-other | 7 | 7 | 7 | 8 | 15 | 18 | 18 | 10 | 14 | 9 | 16 | 7 | 0.0202* |
| HD-ZIP | 48 | 40 | 31 | 40 | 80 | 81 | 78 | 49 | 52 | 32 | 63 | 33 | 0.0752 |
| HRT-like | 2 | 1 | 2 | 1 | 1 | 1 | 1 | 1 | 3 | 1 | 1 | 1 | 0.0001* |
| HSF | 24 | 22 | 17 | 35 | 39 | 40 | 37 | 21 | 26 | 18 | 30 | 19 | 0.1507 |
| LBD | 43 | 47 | 34 | 37 | 68 | 77 | 66 | 65 | 61 | 42 | 58 | 44 | 0.4287 |
| LFY | 1 | 1 | 1 | 1 | 1 | 1 | 1 | 2 | 1 | 1 | 1 | 1 | <0.0001* |
| LSD | 3 | 4 | 3 | 3 | 6 | 9 | 8 | 3 | 4 | 3 | 5 | 3 | 0.0032* |
| M-type_MADS | 66 | 19 | 37 | 54 | 79 | 42 | 35 | 55 | 97 | 49 | 54 | 18 | 0.7617 |
| MIKC_MADS | 42 | 26 | 21 | 42 | 51 | 60 | 53 | 40 | 38 | 33 | 51 | 36 | 0.9598 |
| MYB | 144 | 115 | 98 | 164 | 235 | 260 | 208 | 118 | 176 | 124 | 213 | 138 | 0.4613 |
| MYB_related | 66 | 57 | 61 | 65 | 89 | 101 | 129 | 87 | 102 | 63 | 101 | 57 | 0.0870 |
| NAC | 113 | 84 | 108 | 164 | 153 | 148 | 139 | 97 | 97 | 115 | 170 | 71 | 0.6155 |
| NF-X1 | 2 | 2 | 2 | 3 | 2 | 2 | 3 | 2 | 3 | 2 | 3 | 3 | 0.0002* |
| NF-YA | 10 | 7 | 6 | 8 | 16 | 11 | 11 | 8 | 8 | 6 | 13 | 7 | 0.1321 |
| NF-YB | 13 | 15 | 13 | 16 | 24 | 25 | 18 | 66 | 23 | 14 | 21 | 17 | 0.0001* |
| NF-YC | 14 | 8 | 7 | 8 | 14 | 23 | 18 | 11 | 12 | 9 | 18 | 8 | 0.1444 |
| NZZ/SPL | 1 | 0 | 2 | 0 | 1 | 2 | 2 | 0 | 0 | 2 | 4 | 1 | 0.0461* |
| Nin-like | 14 | 8 | 8 | 8 | 20 | 14 | 12 | 14 | 13 | 8 | 20 | 8 | 0.0198* |
| RAV | 6 | 4 | 4 | 4 | 9 | 7 | 3 | 3 | 3 | 5 | 4 | 1 | 0.3423 |
| S1Fa-like | 3 | 2 | 1 | 1 | 4 | 2 | 2 | 3 | 3 | 1 | 2 | 2 | 0.1230 |
| SAP | 1 | 1 | 1 | 1 | 2 | 2 | 6 | 2 | 1 | 1 | 1 | 1 | <0.0001* |
| SBP | 17 | 15 | 15 | 16 | 30 | 32 | 30 | 16 | 24 | 17 | 30 | 19 | 0.0099* |
| SRS | 11 | 9 | 6 | 7 | 13 | 16 | 16 | 7 | 11 | 6 | 10 | 5 | 0.2371 |
| STAT | 2 | 1 | 1 | 0 | 1 | 1 | 1 | 1 | 1 | 1 | 2 | 1 | 0.0008* |
| TALE | 21 | 20 | 18 | 17 | 44 | 35 | 27 | 22 | 23 | 20 | 34 | 21 | 0.0224* |
| TCP | 24 | 27 | 16 | 16 | 38 | 35 | 23 | 24 | 21 | 20 | 37 | 15 | 0.1323 |
| Trihelix | 29 | 28 | 32 | 24 | 51 | 48 | 43 | 36 | 36 | 34 | 59 | 26 | 0.3505 |
| VOZ | 2 | 2 | 2 | 3 | 3 | 4 | 2 | 2 | 2 | 2 | 4 | 2 | 0.0004* |
| WOX | 16 | 11 | 11 | 9 | 20 | 23 | 21 | 14 | 19 | 10 | 18 | 11 | 0.2291 |
| WRKY | 72 | 62 | 52 | 79 | 120 | 147 | 137 | 65 | 108 | 58 | 102 | 59 | 0.1082 |
| Whirly | 3 | 2 | 2 | 2 | 2 | 4 | 3 | 2 | 3 | 2 | 3 | 2 | 0.0017* |
| YABBY | 6 | 8 | 8 | 6 | 12 | 16 | 10 | 8 | 8 | 6 | 12 | 7 | 0.0369* |
| ZF-HD | 17 | 13 | 12 | 10 | 24 | 24 | 26 | 22 | 18 | 10 | 21 | 10 | 0.1156 |
| bHLH | 153 | 130 | 114 | 145 | 225 | 229 | 203 | 133 | 182 | 129 | 202 | 115 | 0.1097 |
| bZIP | 74 | 62 | 51 | 63 | 115 | 140 | 120 | 62 | 82 | 52 | 96 | 47 | 0.1275 |
| total | 1726 | 1408 | 1349 | 1735 | 2667 | 2767 | 2466 | 1701 | 2152 | 1510 | 2488 | 1276 |  |

*: P < 0.05

### Table S10. The Z-score for gene number of each transcription factor family in *M. candidum* and 11 other plant species

| Family | *Arabidopsis thaliana* | *Cucumis sativus* | *Cirtus sinensis* | *Eucalyptus grandis* | *Gossypium raimondii* | *Melastoma candidum* | *Melastoma dodecandrum* | *Mimulus guttatus* | *Medicago truncatula* | *Prunus persica* | *Populus trichocarpa* | *Vitis vinifera* |
| --- | --- | --- | --- | --- | --- | --- | --- | --- | --- | --- | --- | --- |
| AP2 | -0.9618 | -0.9618 | -1.3169 | -0.2515 | 1.5240 | 1.1689 | -0.0740 | 0.4587 | 0.6362 | -0.7842 | 1.3465 | -0.7842 |
| B3 | 0.1476 | -1.2527 | -0.7997 | 0.9713 | 0.7242 | -0.1819 | -0.5938 | -0.6761 | 0.8065 | 0.2711 | 1.9597 | -1.3763 |
| C2H2 | -0.3283 | -0.6669 | -0.9747 | -0.1744 | 1.5185 | 1.4569 | 1.0568 | -0.3283 | -0.2052 | -0.8824 | 0.9644 | -1.4364 |
| C3H | -0.2051 | -1.2373 | -0.9197 | -0.9991 | 1.7798 | 1.3828 | 0.9065 | -0.6815 | 0.1919 | -0.2051 | 0.7477 | -0.7609 |
| Dof | -0.2355 | -0.2355 | -0.9419 | -0.8242 | 1.1774 | 1.6484 | 1.6484 | -0.7064 | 0.1177 | -0.8830 | 0.2943 | -1.0597 |
| ERF | -0.5054 | -0.5650 | -0.9031 | -0.3861 | 1.6222 | 1.5029 | 0.8467 | -0.5849 | 0.7274 | -0.9230 | 0.5286 | -1.3604 |
| FAR1 | -0.9451 | -0.7361 | 0.2047 | 0.2047 | -0.0044 | -1.2064 | -0.6315 | -0.1612 | 2.1908 | 1.1455 | 0.8319 | -0.8929 |
| GRAS | -1.0265 | -0.9419 | -0.7164 | 0.4111 | 0.3265 | 1.9614 | 1.3131 | -0.8292 | -0.0963 | -0.6319 | 1.0030 | -0.7728 |
| HD-ZIP | -0.2247 | -0.6476 | -1.1233 | -0.6476 | 1.4670 | 1.5198 | 1.3612 | -0.1718 | -0.0132 | -1.0705 | 0.5683 | -1.0176 |
| HSF | -0.3907 | -0.6251 | -1.2112 | 0.8986 | 1.3675 | 1.4847 | 1.1330 | -0.7423 | -0.1563 | -1.0940 | 0.3126 | -0.9768 |
| LBD | -0.7502 | -0.4644 | -1.3932 | -1.1788 | 1.0360 | 1.6790 | 0.8931 | 0.8216 | 0.5358 | -0.8216 | 0.3215 | -0.6787 |
| M-type_MADS | 0.6784 | -1.3676 | -0.5840 | 0.1560 | 1.2443 | -0.3664 | -0.6711 | 0.1995 | 2.0278 | -0.0617 | 0.1560 | -1.4111 |
| MIKC_MADS | 0.0804 | -1.3225 | -1.7609 | 0.0804 | 0.8695 | 1.6586 | 1.0448 | -0.0950 | -0.2703 | -0.7087 | 0.8695 | -0.4457 |
| MYB | -0.4212 | -0.9744 | -1.2986 | -0.0397 | 1.3145 | 1.7914 | 0.7995 | -0.9171 | 0.1892 | -0.8027 | 0.8949 | -0.5357 |
| MYB_related | -0.6637 | -1.0491 | -0.8778 | -0.7065 | 0.3212 | 0.8350 | 2.0340 | 0.2355 | 0.8778 | -0.7922 | 0.8350 | -1.0491 |
| NAC | -0.2642 | -1.1567 | -0.4181 | 1.3055 | 0.9669 | 0.8130 | 0.5360 | -0.7566 | -0.7566 | -0.2026 | 1.4901 | -1.5568 |
| TCP | -0.0821 | 0.2875 | -1.0678 | -1.0678 | 1.6427 | 1.2731 | -0.2053 | -0.0821 | -0.4518 | -0.5750 | 1.5195 | -1.1910 |
| Trihelix | -0.7490 | -0.8407 | -0.4739 | -1.2076 | 1.2687 | 0.9936 | 0.5350 | -0.1070 | -0.1070 | -0.2904 | 2.0025 | -1.0242 |
| WRKY | -0.4954 | -0.7972 | -1.0990 | -0.2842 | 0.9531 | 1.7679 | 1.4661 | -0.7067 | 0.5910 | -0.9179 | 0.4099 | -0.8877 |
| ZF-HD | -0.0410 | -0.6976 | -0.8618 | -1.1901 | 1.1080 | 1.1080 | 1.4363 | 0.7797 | 0.1231 | -1.1901 | 0.6156 | -1.1901 |
| bHLH | -0.2426 | -0.7826 | -1.1583 | -0.4304 | 1.4479 | 1.5418 | 0.9313 | -0.7122 | 0.4383 | -0.8061 | 0.9078 | -1.1348 |
| bZIP | -0.2063 | -0.5971 | -0.9553 | -0.5645 | 1.1290 | 1.9432 | 1.2919 | -0.5971 | 0.0543 | -0.9228 | 0.5102 | -1.0856 |

### Table S11. Summary of genome and transcriptome sequencing data of *Melastoma candidum*

| **Sequencing platform** | **Library type** | **Number of nucleotides** | **Depth** | **Average length** |
| --- | --- | --- | --- | --- |
| PacBio | DNA-seq | 43,607,829,756 | 170× | 19,248 |
| Illumina | DNA-seq | 10,938,816,500 | 42× | 125 |
| Illumina | DNA-seq | 68,888,815,800 | 269× | 150 |
| Illumina | RNA-seq(leaf) | 38,317,894 | - | 125 |
|  | RNA-seq(fruit) | 37,346,062 | - | 125 |
|  | RNA-seq(branch) | 42,255,228 | - | 125 |
|  | RNA-seq(flower) | 38,509,596 | - | 125 |
|  | RNA-seq (stamen-outer whorl) | 38,537,698 | - | 125 |
|  | RNA-seq (stamen-inner whorl) | 38,859,532 |  | 125 |

### Table S12. Sources of genome and transcriptome data of 12 species included in the study

| **Species** | **Type** | **Family** | **Order** | **Source (Accession ID of NCBI)** | **Citations** | **URLs** |
| --- | --- | --- | --- | --- | --- | --- |
| *Arabidopsis thaliana* | genome | Brassicaceae | Brassicales | Phytozome | (Lamesch, et al. 2011) | https://phytozome-next.jgi.doe.gov/info/Athaliana_TAIR10 |
| *Cirtus sinensis* | genome | Rutaceae | Sapindales | Phytozome (JJOQ01000000) | (Wu, et al. 2014) | https://phytozome-next.jgi.doe.gov/info/Csinensis_v1_1 |
| *Cucumis sativus* | genome | Cucurbitaceae | Cucurbitales | Phytozome | - | https://phytozome-next.jgi.doe.gov/info/Csativus_v1_0 |
| *Eucalyptus grandis* | genome | Myrtaceae | Myrtales | Phytozome | (Myburg, et al. 2014) | https://phytozome-next.jgi.doe.gov/info/Egrandis_v2_0 |
| *Gossypium raimondii* | genome | Malvaceae | Malvales | Phytozome (ALYE01000000) | (Paterson, et al. 2012) | https://phytozome-next.jgi.doe.gov/info/Graimondii_v2_1 |
| *Medicago truncatula* | genome | Fabaceae | Fabales | Phytozome | (Tang, et al. 2014) | https://phytozome-next.jgi.doe.gov/info/Mtruncatula_Mt4_0v1 |
| *Melastoma dodecandrum* | genome | Melastomataceae | Myrtales | provided by authors of this paper | (Hao, et al. 2022) | provided by authors of this paper |
| *Mimulus guttatus* | genome | Phrymaceae | Lamiales | Phytozome | (Hellsten, et al. 2013) | https://phytozome-next.jgi.doe.gov/info/Mguttatus_v2_0 |
| *Osbeckia opipara* | transcriptome | Melastomataceae | Myrtales | This study (GKED00000000) | This study | This study |
| *Populus trichocarpa* | genome | Salicaceae | Malpighiales | Phytozome (AARH02000000) | (Tuskan, et al. 2006) | https://phytozome-next.jgi.doe.gov/info/Ptrichocarpa_v3_0 |
| *Prunus persica* | genome | Rosaceae | Rosales | Phytozome (AKXU02000000) | (Verde, et al. 2013) | https://phytozome-next.jgi.doe.gov/info/Ppersica_v2_1 |
| *Vitis vinifera* | genome | Vitaceae | Vitales | Phytozome | (Jaillon, et al. 2007) | https://phytozome-next.jgi.doe.gov/info/Vvinifera_v2_1 |

# Supplementary Figures

**
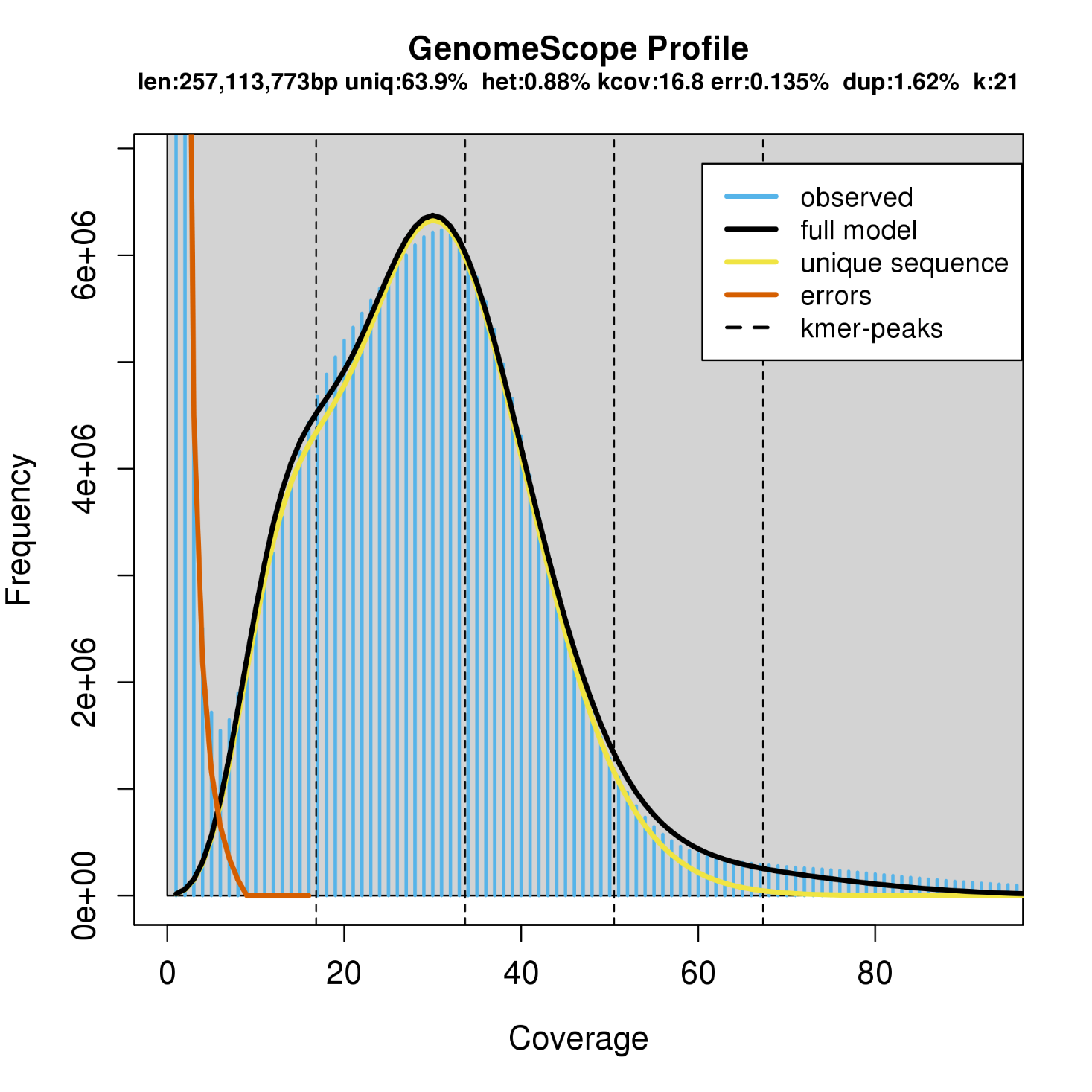
**

### Figure S1. Estimation of genome size of *Melastoma candidum* using K-mer statistics (k = 21). The counts of 21-mer were calculated using jellyfish (version2.3.0) with clean reads from short insert size libraries, and the genome size was estimated to be 257.1Mb using GenomeScope1.0.


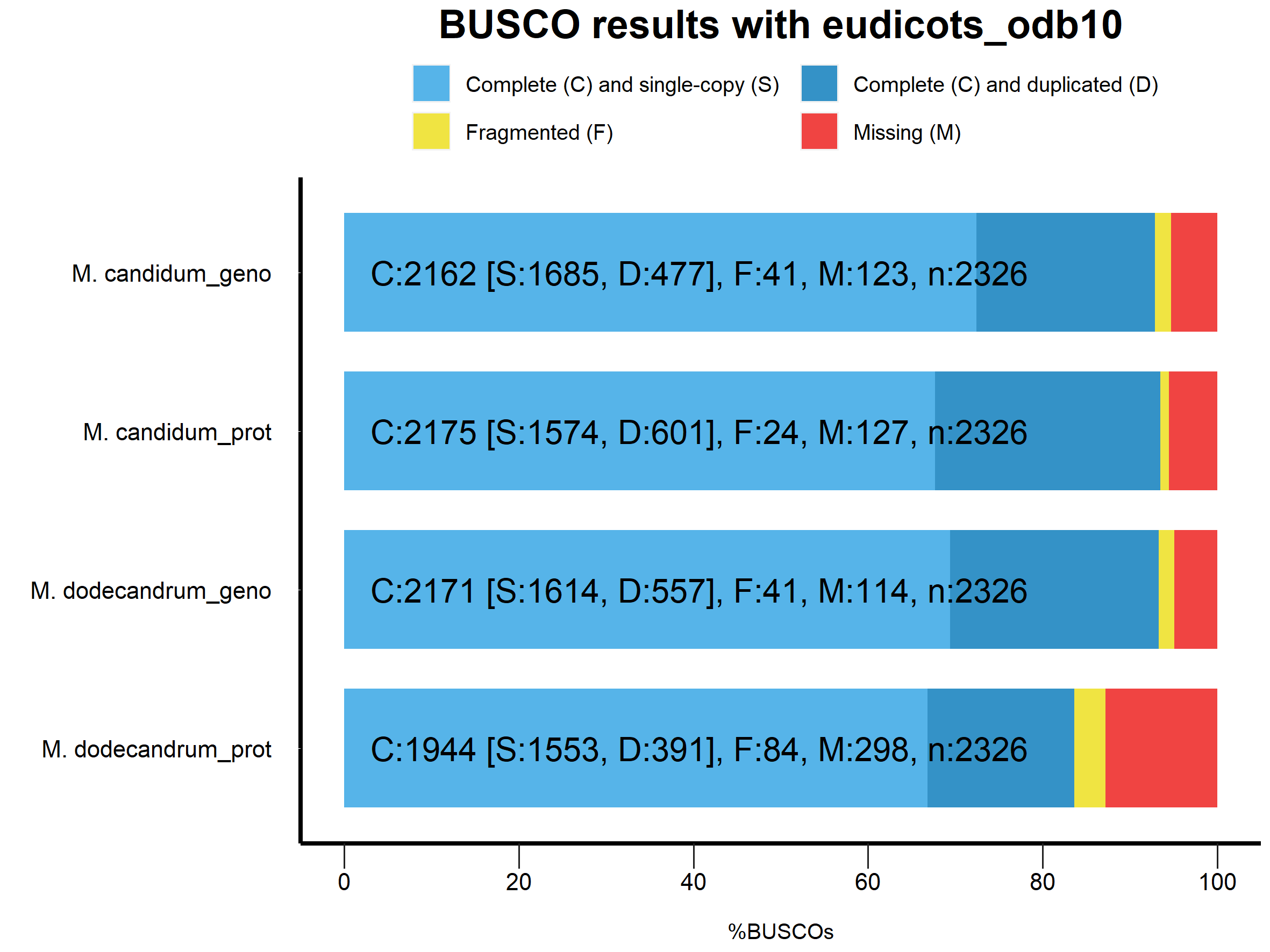


### Figure S2. BUSCO assessment of the genome assemblies and proteomes of *Melastoma candidum* and *M. dodecandrum* with the eudicots_odb10 dataset.


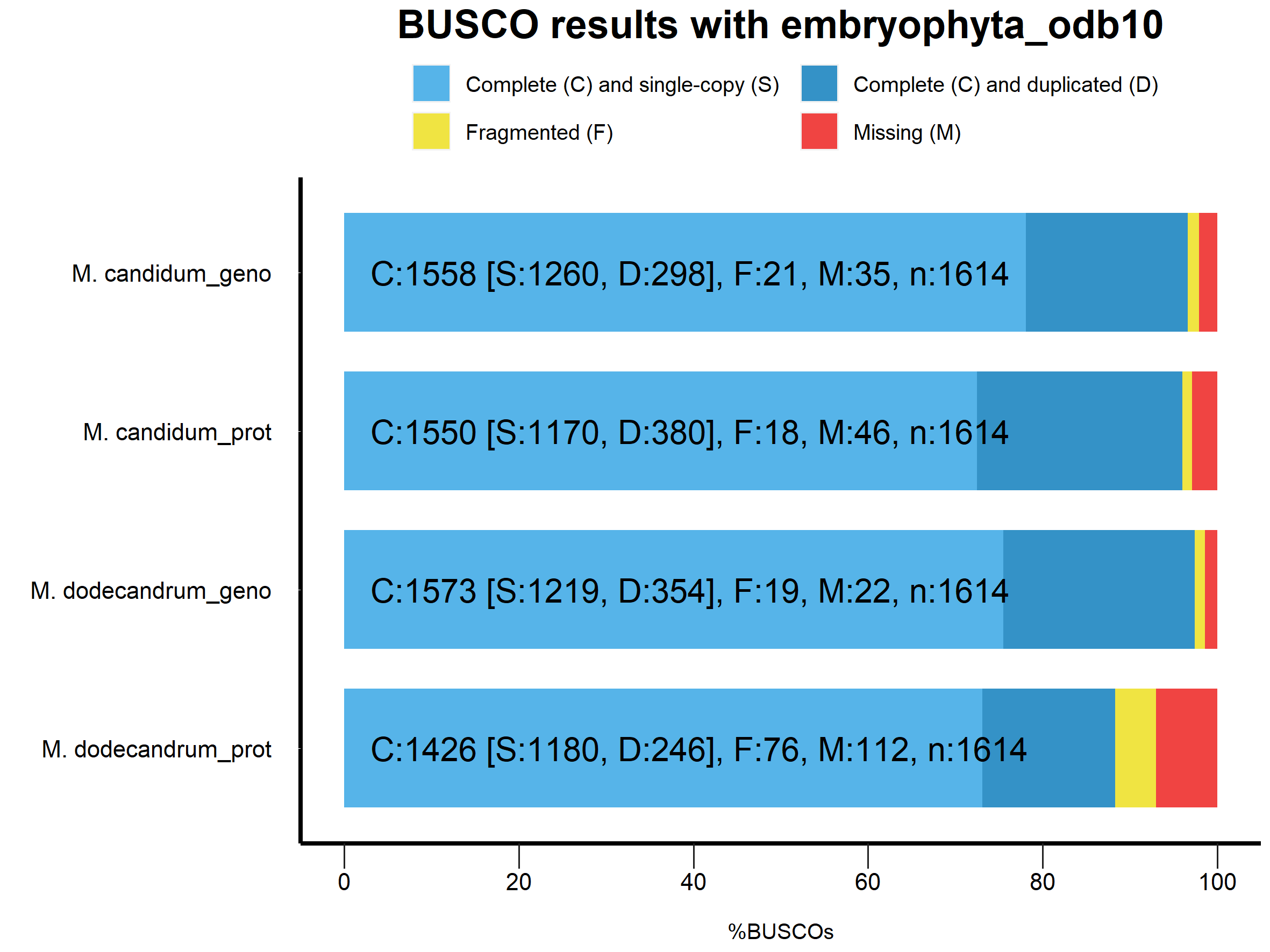


### Figure S3. BUSCO assessment of the genome assemblies and proteomes of *Melastoma candidum* and *M. dodecandrum* with the embryophyta_odb10 dataset.

**
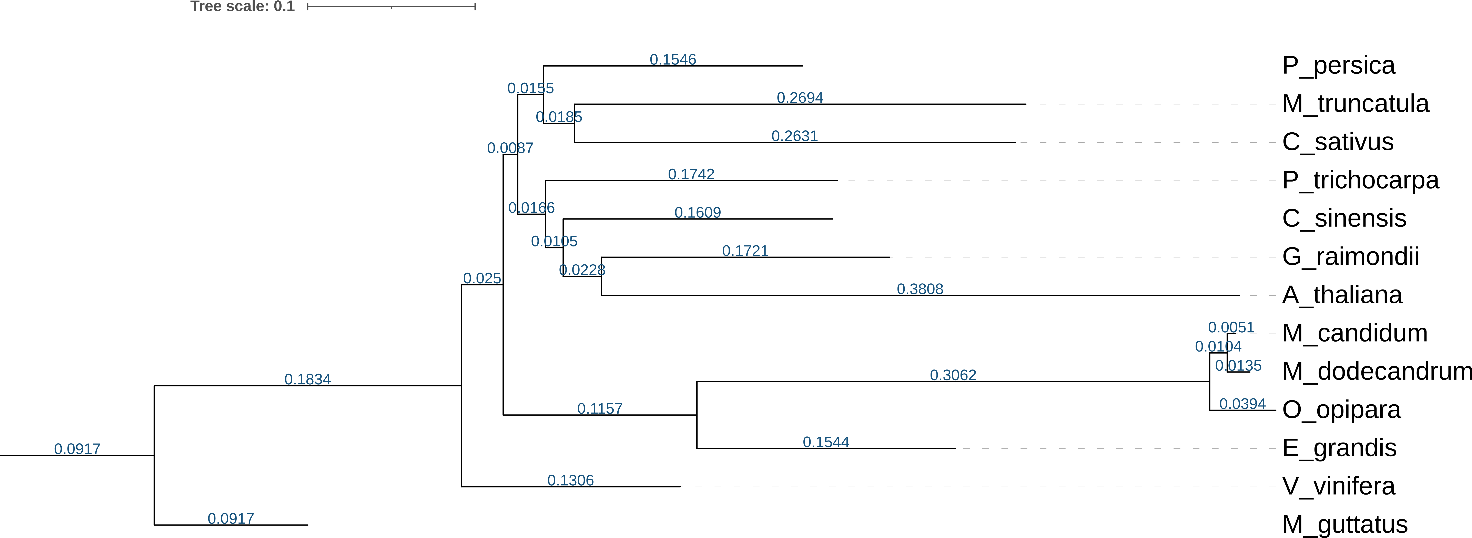
**

### Figure S4. The maximum likelihood tree of 13 plant species based on concatenated sequences of 346 single-copy genes. Branch length was shown on each branch.

Species abbreviations: A_thaliana: *Arabidopsis thaliana*; C_sinensis: *Cirtus sinensis*; C_sativus: *Cucumis sativus*; E_grandis: *Eucalyptus grandis*; G_raimondii: *Gossypium raimondii*; M_truncatula: *Medicago truncatula*; M_dodecandrum: *Melastoma dodecandrum*; M_candidum: *M. candidum*; O_opipara: *Osbeckia opipara*; P_trichocarpa: *Populus trichocarpa*; P_persica: *Prunus persica*; V_vinifera: *Vitis vinifera*; M_guttatus: *Mimulus guttatus*.


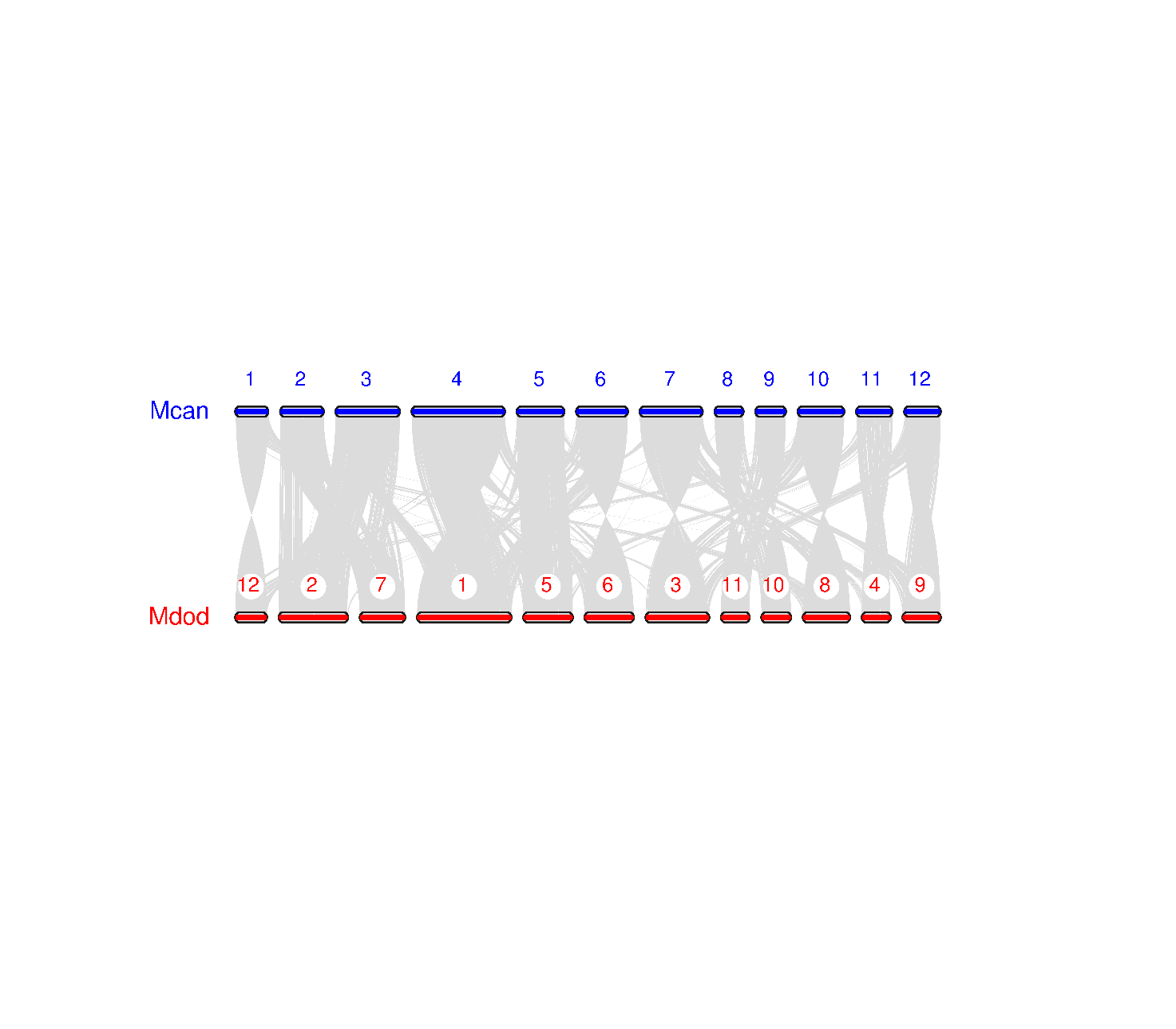


### Figure S5. Genome synteny analysis between *Melastoma candidum* and *M. dodecandrum*.

Species abbreviations: Mcan: *Melastoma candidum*; Mdod: *M. dodecandrum*.


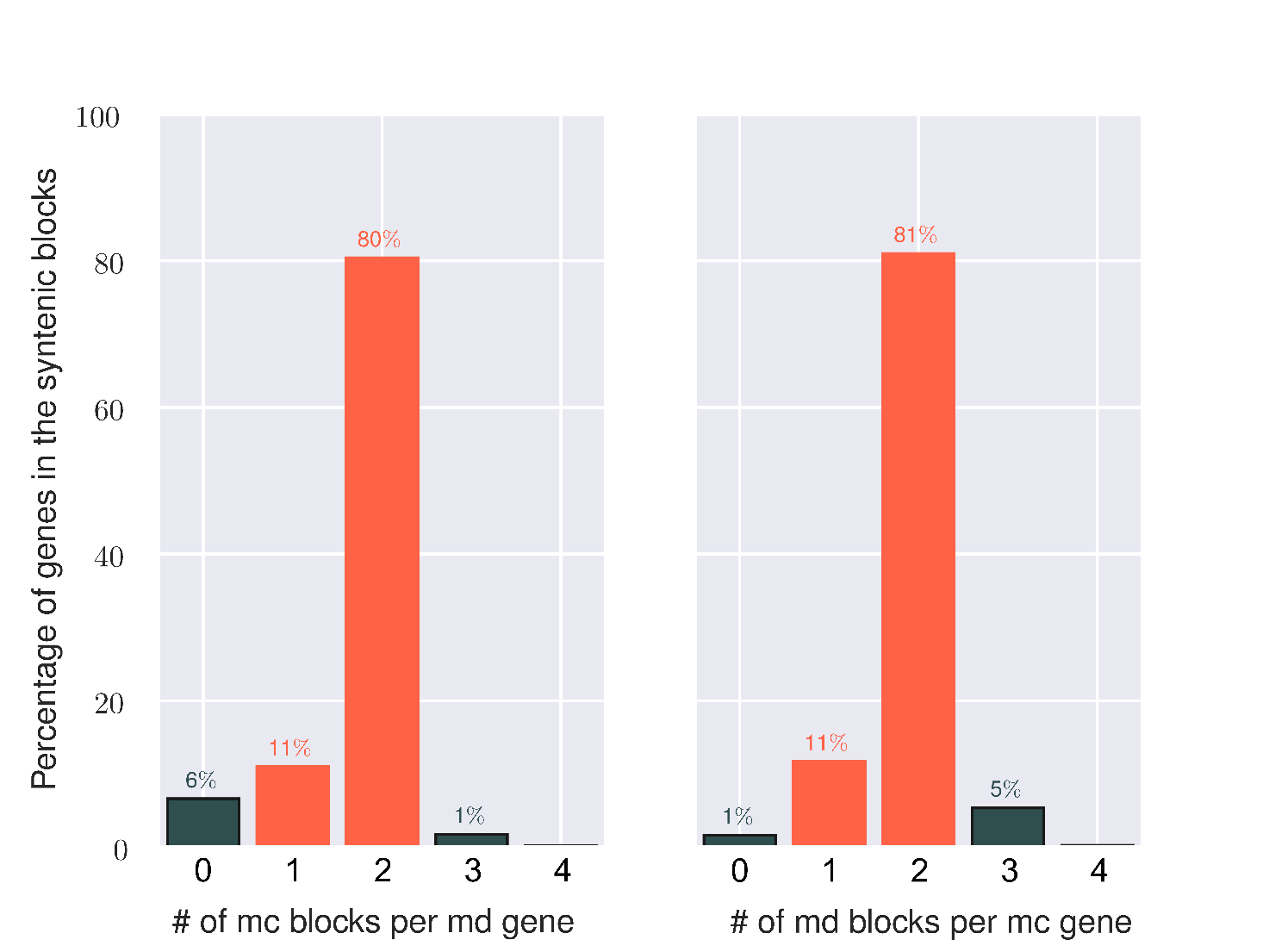


### Figure S6. Syntenic depths of blocks per gene between *Melastoma candidum* and *M. dodecandrum*

mc: *Melastoma candidum*; md: *M. dodecandrum*


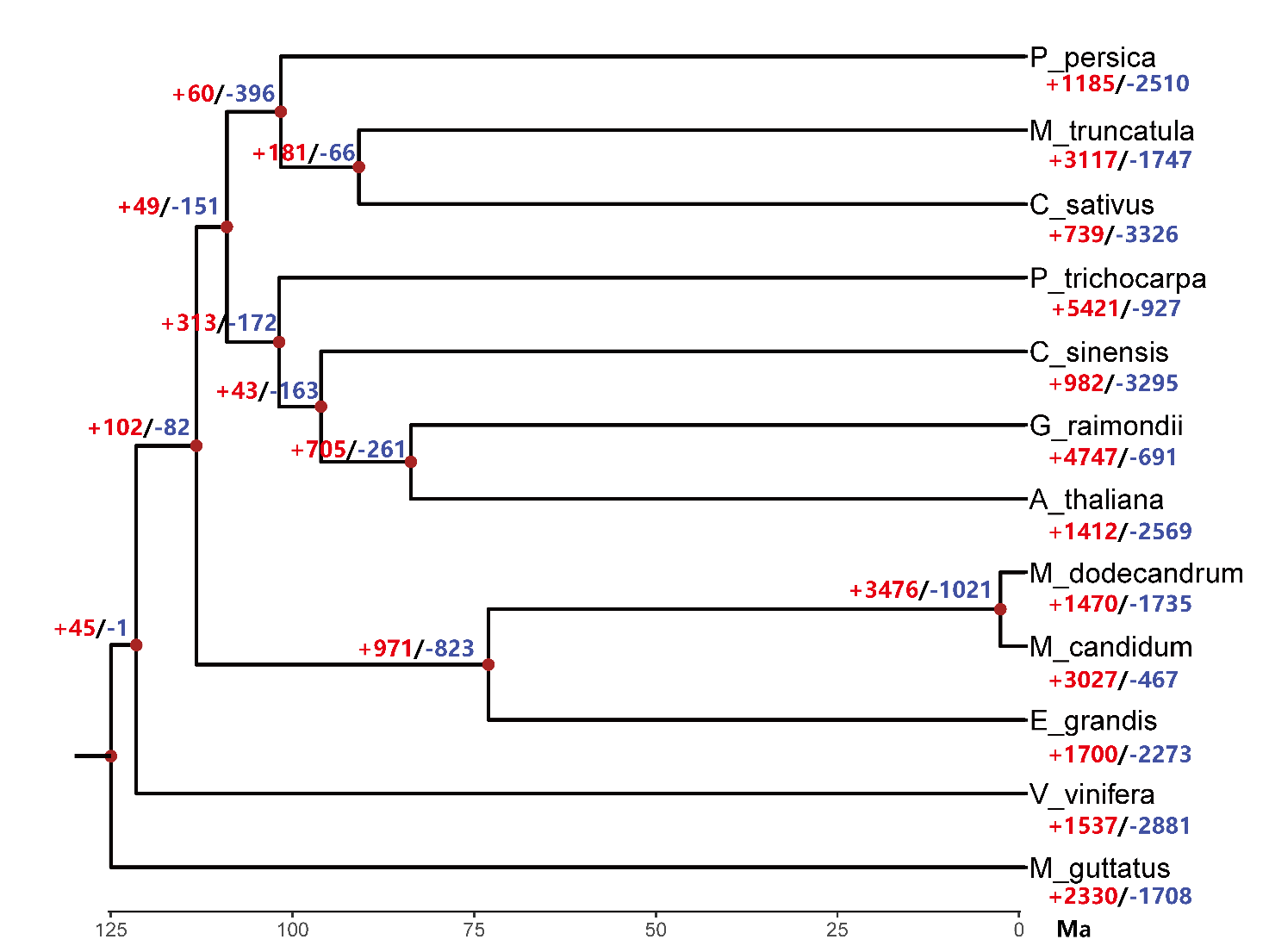


### Figure S7. Gene family expansion and contraction in 12 plant species including two species of *Melastoma*. The chronogram tree is constructed based on concatenated sequences of 601 single-copy genes. Numbers of expanded (in red) and contracted (in blue) gene families are shown on each node. All nodes have 100% bootstrap support.

Species abbreviations: A_thaliana: *Arabidopsis thaliana*; C_sinensis: *Cirtus sinensis*; C_sativus: *Cucumis sativus*; E_grandis: *Eucalyptus grandis*; G_raimondii: *Gossypium raimondii*; M_truncatula: *Medicago truncatula*; M_dodecandrum: *Melastoma dodecandrum*; M_candidum: *M. candidum*; P_trichocarpa: *Populus trichocarpa*; P_persica: *Prunus persica*; V_vinifera: *Vitis vinifera*; M_guttatus: *Mimulus guttatus*.

# References

Hao Y, et al. 2022. The *Melastoma dodecandrum* genome and the evolution of Myrtales. Journal of Genetics and Genomics 49: 120-131. doi: 10.1016/j.jgg.2021.10.004

Hellsten U, et al. 2013. Fine-scale variation in meiotic recombination in *Mimulus* inferred from population shotgun sequencing. Proceedings of the National Academy of Sciences 110: 19478-19482. doi: doi:10.1073/pnas.1319032110

Jaillon O, et al. 2007. The grapevine genome sequence suggests ancestral hexaploidization in major angiosperm phyla. Nature 449: 463-467. doi: 10.1038/nature06148

Lamesch P, et al. 2011. The *Arabidopsis* Information Resource (TAIR): improved gene annotation and new tools. Nucleic Acids Research 40: D1202-D1210. doi: 10.1093/nar/gkr1090

Myburg AA, et al. 2014. The genome of Eucalyptus grandis. Nature 510: 356-362. doi: 10.1038/nature13308

Paterson AH, et al. 2012. Repeated polyploidization of *Gossypium* genomes and the evolution of spinnable cotton fibres. Nature 492: 423-427. doi: 10.1038/nature11798

Tang H, et al. 2014. An improved genome release (version Mt4.0) for the model legume Medicago truncatula. BMC Genomics 15: 312. doi: 10.1186/1471-2164-15-312

Tuskan GA, et al. 2006. The Genome of Black Cottonwood, *Populus trichocarpa*. Science 313: 1596-1604. doi: doi:10.1126/science.1128691

Verde I, et al. 2013. The high-quality draft genome of peach (*Prunus persica*) identifies unique patterns of genetic diversity, domestication and genome evolution. Nature Genetics 45: 487-494. doi: 10.1038/ng.2586

Wu GA, et al. 2014. Sequencing of diverse mandarin, pummelo and orange genomes reveals complex history of admixture during citrus domestication. Nature Biotechnology 32: 656-662. doi: 10.1038/nbt.2906
